# A novel ViT-BILSTM model for physical activity intensity classification in adults using gravity-based acceleration

**DOI:** 10.1186/s42490-025-00088-2

**Published:** 2025-02-01

**Authors:** Lin Wang, Zizhang Luo, Tianle Zhang

**Affiliations:** 1 Faculty of Health and Life Sciences, University of Exeter, Heavitree Road, Exeter, EX1 2LU UK; 2 Engineering & Technology College, Yangtze University, Jingzhou, 434023 China; 3 Department of Computer Science, University of Liverpool, Liverpool, L69 3DR UK

**Keywords:** Deep learning, Raw accelerometer data, Variation, Generalisation, Physical activity patterns

## Abstract

**Aim:**

The aim of this study is to apply a novel hybrid framework incorporating a Vision Transformer (ViT) and bidirectional long short-term memory (Bi-LSTM) model for classifying physical activity intensity (PAI) in adults using gravity-based acceleration. Additionally, it further investigates how PAI and temporal window (TW) impacts the model’ s accuracy.

**Method:**

This research used the Capture-24 dataset, consisting of raw accelerometer data from 151 participants aged 18 to 91. Gravity-based acceleration was utilised to generate images encoding various PAIs. These images were subsequently analysed using the ViT-BiLSTM model, with results presented in confusion matrices and compared with baseline models. The model’s robustness was evaluated through temporal stability testing and examination of accuracy and loss curves.

**Result:**

The ViT-BiLSTM model excelled in PAI classification task, achieving an overall accuracy of 98.5% ± 1.48% across five TWs-98.7% for 1s, 98.1% for 5s, 98.2% for 10s, 99% for 15s, and 98.65% for 30s of TW. The model consistently exhibited superior accuracy in predicting sedentary (98.9% ± 1%) compared to light physical activity (98.2% ± 2%) and moderate-to-vigorous physical activity (98.2% ± 3%). ANOVA showed no significant accuracy variation across PAIs (F = 2.18, *p* = 0.13) and TW (F = 0.52, *p* = 0.72). Accuracy and loss curves show the model consistently improves its performance across epochs, demonstrating its excellent robustness.

**Conclusion:**

This study demonstrates the ViT-BiLSTM model’s efficacy in classifying PAI using gravity-based acceleration, with performance remaining consistent across diverse TWs and intensities. However, PAI and TW could result in slight variations in the model’s performance. Future research should concern and investigate the impact of gravity-based acceleration on PAI thresholds, which may influence model’s robustness and reliability.

**Supplementary information:**

The online version contains supplementary material available at 10.1186/s42490-025-00088-2.

## Introduction

The use of accelerometer-based measurements for physical activity (PA) has become increasingly prevalent, as it reduces biases inherent in self-reported data and provides more accurate and insightful information on PA [1, 2]. However, this method presents challenges, particularly in classifying different intensities of PA [3–5]. Different intensities of PA can have varying effects on health. For instance, prolonged periods of light PA (LPA) and moderate-to-vigorous PA (MVPA) have different impacts on cardiovascular health in adults [6, 7]. Thus, accurately capturing different intensities is crucial for understanding their health implications [8–10].

Traditionally, accelerometer data processing methods estimate PA intensity using cut-point that based on metabolic equivalents (METs), such as LPA <3 METs; MPA = 3–5.99 METs; VPA ≥6 METs [11]. Alternatively, studies have used counts, which represent the cumulative acceleration signals within a specified time interval (epoch), typically filtered to remove noise and high-frequency vibrations, and expressed as an aggregate value for each epoch [12]. Within the adults population, various studies use different step count thresholds to classify LPA, MVPA, and sedentary (SB), such as, for MVPA, thresholds include ≥1952 counts per minute [12], and ≥2020 counts/min [13]. Using different cut points for the same population and the same intensity complicates comparative analyses. Furthermore, METs-based intensity estimation can be affected by individual differences, environmental factors, and device placement, leading to inaccuracies [14].

Recent research has explored machine learning methods to overcome the limitations of cut points in classifying PA intensities [15–18]. Previous work predominantly relied on traditional machine learning algorithms like k-Nearest Neighbours (*k–NN*), Support Vector Machine (SVM), Random Forest (RF), hidden semi-Markov models for activity recognition [17, 19, 20]. These machine learning methods have shown good performance and efficiency in classifying PA intensities. However, they depend on manually designed and selected features, which are time-consuming and may miss important features [9, 16, 21]. For example, Chong, Tjurin [22] used filter, wrapper, and embedded methods to find suitable feature subsets for activity prediction. While wrappers can find better feature subsets, they are computationally expensive and prone to overfitting. Filter and wrapper methods also struggle to capture complex feature interactions. Consequence, convolutional neural networks (CNNs) have emerged as a powerful alternative due to their ability to automatically learn and extract relevant features from raw data without manual intervention. This characteristic enables CNNs to capture complex patterns and interactions within the data that traditional machine learning methods might miss.

The study by Nawaratne, Alahakoon [16] represents an advancement in the field of accelerometer-based PA by leveraging deep learning, the research provides a more accurate, user-friendly approach to predicting energy expenditure and physical activity intensity in free-living conditions. Specifically, the Convolutional Neural Network with custom feature extraction models for Untrained Group results shows that SB achieved correct predictions of 85.4%, LPA achieved correct predictions of 84.2% and MVPA achieved correct predictions of 63.1%. The study by Widianto, Sugiarto [21], applied a CNN model to classify PAI in adults wearing five accelerometers. The model achieved accuracies of 97% for MPA, 95% for LPA, and 98% for SD. However, previous studies were conducted in laboratory settings. The CNN model, which demonstrated excellent performance, required participants to wear five accelerometers. This setup reduces the feasibility of capturing physical activity in natural environments. Moreover, these studies rely on unidimensional time-series data, potentially failing to capture the global information during activities.

Recently, Farrahi, Muhammad [15] applied AccNet24 framework that has laid the groundwork for analysing 24-h PA behaviours using wrist-worn accelerometer data in free-living conditions, applied recurrent neural networks (RNNs), including BiLSTM (Bidirectional Long Short-Term Memory) networks to classify PAI. Unlike traditional one-dimensional raw accelerometer data processing, this framework uses two-dimensional (2D) images to handle the data. This approach can provide the model with richer information. Moreover, BiLSTM networks excel at processing time-series data by capturing dynamic patterns and temporal dependencies in both forward and backward directions. This bidirectional architecture allows the network to utilize information from both past and future states at each time step, which is particularly valuable for time-series data where the context of both preceding and succeeding data points can influence the interpretation of a given point [23]. This is particularly useful when processing continuous activity data or transitions between different activities. Nevertheless, this model is not sensitive to the global spatial information of the activity, meaning that intensity information might be easily overlooked. On the other hand, ViT excels at extracting complex spatial features from images, such as changes in motion position and intensity, as well as, the ViT model leverages a global attention mechanism to identify more meaningful global features within the images, further enhancing classification performance [24]. Consequently, this study combines the strengths of ViT and BiLSTM, fully leveraging the spatial features of images and the temporal features of time series to improve the accuracy of activity intensity classification. Furthermore, temporal stability is a crucial factor affecting model performance, especially for images generated from time-series data [25]. Farrahi, Muhammad [15] study just a 30-s TW to generate images; it may overlook that TW is a crucial factor influencing PA features in adults, consequently affecting the model’s accuracy. Meanwhile, the variance in intensity might also influence model’s accuracy as SD typically involves longer, stable periods, whereas MVPA requires higher gravity acceleration and is more distinct. But, predicting LPA is more challenging due to its complexity, such as slow walking, fast walking, or occasional movement [26]. Additionally, Farrahi, Muhammad [15] used METs-based classification from video-labelled datasets, which may limit the model’s practical utility. As METs-based activity intensity has been shown to confuse intensity types [14], further affecting the connection between intensity and health outcomes [6, 7]. Recent studies advocate the use of gravity-based acceleration to cumulate PA, as it can reduce errors associated with traditional methods such as cut points and METs in calculating accumulated activity [5, 27].

Therefore, this study used the ViT-BiLSTM model to classify images encoded from gravity-based acceleration data to determine PAI, Further, it considers how temporal stability (different TWs for images), and PAI, affect the model’s accuracy. The objectives of this study are: (1) to use the ViT-BiLSTM model to predict PA intensities calculated from gravity-based acceleration data in adults; (2) to examine the model’s robustness across different TWs and PAIs; and (3) to observation how different PAIs and TWs impact the accuracy of model.

## Methodology

**Fig. 1:**
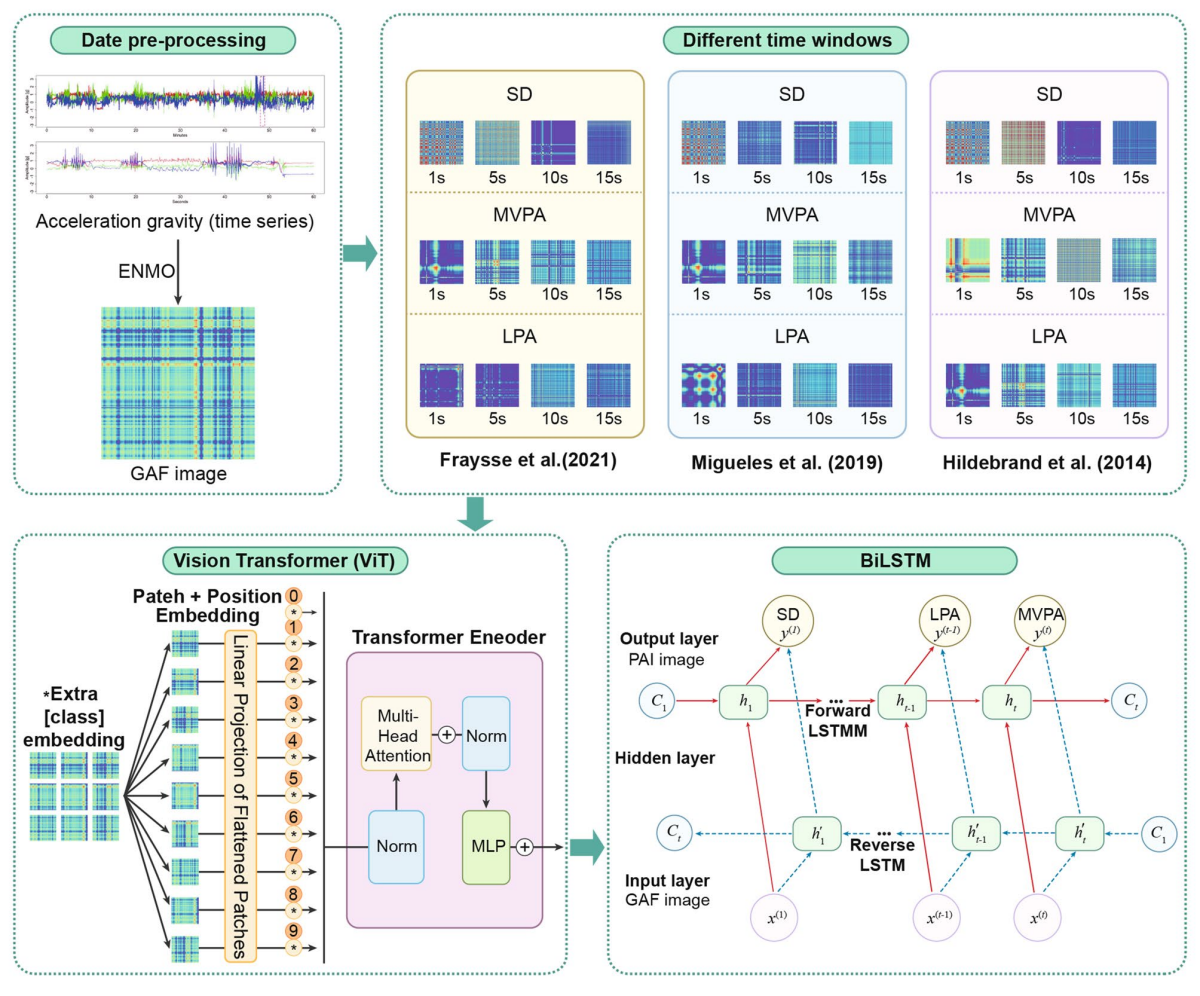
Overall flowchart of ViT-BiLSTM model for physical activity intensity in adults using gravity-based acceleration

In this study, we developed a novel framework to classify PAI employing a hybrid Vision Transformer (ViT) and bidirectional long short-term memory (Bi-LSTM) network. We used raw accelerometer data from 151 participants, which were pre-processed into gravitational acceleration using the Euclidean Norm Minus One (ENMO) algorithm and subsequently converted into GAF images. The overall workflow is illustrated in Fig. 1. Initially, GAF images were generated from the pre-processed data. These images were processed by the ViT component to extract spatial features. These features were subseqently fed into a BiLSTM network to capture temporal dependencies. Finally, a fully connected layer that classified the PAIs into SD, LPA, and MVPA is added on the top of the network.

### Dataset

The present study employed the Capture-24 dataset, which comprises data from Axivity AX3 wrist-worn activity trackers collected from 151 adults aged 18–91 years in Oxfordshire between 2014 and 2016. Participants wore these devices continuously over approximately 24 h at a sampling frequency of 100 Hz, resulting in nearly 4000 h of data, with over 2500 h annotated based on validated ground truth activities [28].

### Participants

The Capture-24 dataset includes 131 participants (78 women): 74 young adults (18–39 years; 32 men, 42 women), 42 middle-aged adults (40–59 years; 7 men, 27 women), and 15 older adults (60+ years; 14 men, 9 women) [29].

### Acceleration signal-to-image

#### Data pre-processing (physical activity intensity labelling)

The Euclidean Norm Minus One (ENMO) algorithm calculates raw gravitational acceleration (g) by subtracting 1 g (1 g = 9.81 m/s^2^) from the Euclidean norm of the three-axis acceleration signals. Its simplicity and effectiveness lie in its ability to separate gravitational and movement components without requiring complex frequency filtering, thereby demonstrating robust performance, particularly in free-living conditions [30]. Subsequently, images are labelled based on the Hildebrand et al. [31] thresholds derived from the raw gravitational acceleration (g).

Equation (1) defines the ENMO, which is used to compute the magnitude of acceleration in second-by-second time series and classify the PAI:

1 $$ENMO = \sqrt {{x^2} + {y^2} + {z^2}} - 1g$$

where x, y, and z represent the components of acceleration in three dimensions. ENMO is calculated by taking the Euclidean norm (*i.e.,* the length of the acceleration vector) in three-dimensional space and then subtracting 1 g (1 g = 9.81 m/s^2^) (the acceleration due to gravity), yielding a corrected value for activity intensity [30]. The x, y, and z orientations of the Axivity AX3 are explained in Supplemental Material 1.

The PAI threshold defined by Hildebrand et al. [31] for adults (18–65 years) uses raw gravity acceleration (g) as follow:

- Sedentary: 0–10 g/s
- LPA: 10–42 g/s PAI threshold of Hildebrand et al. [31]
- MVPA: >42 g/s

for sedentary behaviour, the acceleration is less than 10 g/s; LPA is between 10 and 42 g/s; for MVPA is above 42 g/s.

Additionally, the collection data are based on an acceleration sampling frequency of 100 Hz, meaning that the defined activity intensities correspond to the cumulative acceleration over specific data points within a given time window. For instance, a 1-s window would include 100 data points, while 5-s, 10-s, and 15-s windows would represent 500, 1000, and 1500 data points, respectively.

#### Gramian angular field

To transform raw acceleration signals into GAF images, we followed a comprehensive process involving several steps, as detailed below:

We computed the magnitude of the raw acceleration signal (*x,y,z*) using the ENMO method to remove the effect of gravity and filter out negative values. The result is a signal sequence *v*_*t*_ at each time point *t*, where: *v*_*t*_ represents the magnitude of acceleration at time *t*.

To ensure consistency across all samples, the signal sequence $\left( {{v_t}} \right)$ was normalized to the range [−1, 1]. The normalization formula is as follows:

$$\widetilde {{v_t}} = \frac{{{v_t} - \min \left( v \right)}}{{\max \left( v \right) - \min \left( v \right)}} \cdot 2 - 1,\forall t$$

Where: ${v_t}{\text{ }}$is the original signal value at time $t;$ $\min \left( v \right)$ and${\text{ max}}\left( v \right)$ represent the minimum and maximum values of the signal, respectively; $\widetilde {{v_t}}$ is the normalized signal value.

The normalized signal $\widetilde {{v_t}}$ is mapped into a polar coordinate system, representing the signal in terms of angles and radii.

*Angle* (*θ*_*t*_)

Each normalized signal value is converted into an angular value using the inverse cosine function:

$${\theta _t} = \arccos \left( {\widetilde {{v_t}}} \right),\quad - 1 \leqslant \widetilde {{v_t}} \leqslant 1$$

The angle encodes the relative amplitude of the signal.

Radius (*r*_*t*_)

$${r_t} = \frac{t}{T},\quad t = 1,2, \ldots ,T$$

The radius represents the normalized time index, preserving the sequential nature of the time series, where *T* is the total length of the signal sequence.

Using the angular values ${\theta _t}$ from the polar representation, the GAF matrix is constructed to capture the temporal dependencies of the time series. The matrix elements are defined as:

$$G\left[ {i,j} \right] = \cos \left( {{\theta _i} + {\theta _j}} \right),\quad i,j = 1,2, \ldots ,T$$

where:

G[*i,j*] represents the cosine of the sum of the angles at time points *i* and *j*; This formula encodes both global and local temporal features of the signal. To facilitate visualization, the GAF matrix values were normalized to the range [0, 1]. And then, the GAF images were generated with a resolution of 224 × 224 pixels, which is a widely adopted standard resolution in computer vision tasks, particularly for models using ViT architecture.

### ViT-BiLSTM model

The ViT-BiLSTM model combines the ViT for spatial feature extraction and BiLSTM for capturing temporal dependencies. Below is the detailed algorithmic description:

#### ViT component

##### Patch embedding

The GAF images derived from accelerometer data are divided into fixed-size patches. Each patch is then flattened and mapped to a lower-dimensional space through a linear projection. Given an input image X∈R^*H×W×C*^ of height *H,* width *W,* and *C* channels, the image is divided into *N* patches, each of size *P* *×* *P*. The resulting patches *x*_*p*_ are linearly transformed into embeddings *z*_*p*_:

$${z_p} = {E_{xp}} + ep$$

where *E* is a learnable embedding matrix and *e*_*p*_ is the positional embedding.

##### Positional encoding

Positional encodings *e*_*p*_ are added to the patch embeddings to retain spatial information. These encodings help the model understand the order and position of patches.

##### Transformer encoder

The transformer encoder consists of multiple layers, each containing a multi-head self-attention mechanism and a position-wise feed-forward network. Each encoder layer updates the patch embeddings as follows:

$$z{\prime _p} = {\rm{LayerNorm}}\left( {{z_p} + {\rm{MultiHeadSelfAttention}}\left( {{z_p}} \right)} \right)$$

$${z_p}^{(i + 1)} = {\rm{ LayerNorm }}\left( {z{\prime _p} + {\rm{FeedForward }}\left( {z{\prime _p}} \right)} \right)$$

where *z*^*(i)*^_*p*_ denotes the patch embeddings after the *i–th* encoder layer.

The features extracted by the ViT component are then fed into a BiLSTM model, which captures temporal dependencies through forward and reverse LSTM layers. This bidirectional processing enhances the model’s ability to learn temporal patterns in both directions, contributing to improved classification accuracy.

### BiLSTM component

#### Sequence processing

The feature vectors from the ViT component, representing spatial features of the input image patches, are fed into a BiLSTM network to capture temporal dependencies. The BiLSTM processes the sequence of feature vectors in both forward and backward directions.

Let *h*_*t*_forward and *h*_*t*_backword be the hidden states of the forward and backward LSTM cells at time step *t*, respectively. The BiLSTM outputs are concatenated:

$${h_t}{\text{forward}} = {\text{ LSTM }}({h_{t - 1}}{\text{forward}},{\text{ }}{{\text{x}}_{\text{t}}},{\text{ }}{{\text{c}}_{t - 1}}{\text{forward}}),{\text{ t}} \in \left[ {0,{\text{T}}} \right]$$

$${h_t}{\text{backward}} = {\text{ LSTM }}({h_{t - 1}}{\text{backward}},{\text{ }}{{\text{x}}_{\text{t}}},{\text{ }}{{\text{c}}_{t - 1}}{\text{backward}}),{\text{ t}} \in \left[ {0,{\text{T}}} \right]$$

$${{\text{H}}_t} = [{h_t}{\text{forward}};{h_t}{\text{backward}}]$$

*Concatenation and Classification:*

$${h_{final}} = [{h_t}\,{\text{forward}};{h_t}\,{\text{backward}}]$$

The final output is computed using a softmax layer:

$${\text{Output}} = {\text{Softmax}}\left( {{W_o}{h_{final}} + {b_o}} \right)$$

### Experimental setup

This study encoding images into three different PAI: SD, LPA, and MVPA, using gravity acceleration. For the GAF image generation, data was collected daily from 8 AM to 10 PM, focusing on the time period when SD, LPA, and MVPA activities predominantly occur during typical waking hours. The ViT-BiLSTM model then predicted the PAI. To enhance the credibility of the model, this study compares several models: CNN, ViT, BiLSTM, ViT-BiLSTM, and CNN-BiLSTM. Consequently, the temporal stability testing, boundary value analysis, and accuracy and loss curves assessed robustness of model. ANOVA tests are used to examine the robustness and reliability of the model’s accuracy across various TWs (e.g., 1s, 5s, 30s), ensuring consistent performance regardless of these variations. Additionally, accuracy and loss values in the training-validation process were utilised to observe changes in accuracy and loss values, based on the TW yielding the highest accuracy results. This approach aids in understanding the model’s learning dynamics and stability over time. Finally, we examined the mean and standard deviation values to understand how PAI and TW affect the model’s accuracy.

### Training details

All experiments are carried out on a workstation with NVIDIA 2080ti GPUs, and the dataset is divided into training (60%), validation (20%) and testing (20%) sets. During the training process, model performance was evaluated on both training and validation sets after each epoch. This allowed us to monitor the model’s learning progress and ensure it generalized well to unseen data. The validation performance served as an important indicator for potential overfitting, helping to optimize the model’s hyperparameters and determine when to stop training. During the training process, model performance was evaluated on both training and validation sets after each epoch. This allowed us to monitor the model’s learning progress and ensure it generalized well to unseen data. The validation performance served as an important indicator for potential overfitting, helping to optimize the model’s hyperparameters and determine when to stop training.

As Table 1 shows, the training process utilised a batch size of 16, with a sequence length of 4, resizing each image resized to 224 × 224 pixels. The model was trained over 10 epochs with a learning rate of 1e-5, and weight decay set to 0.001 to prevent overfitting. The selection of 10 training epochs was determined through extensive preliminary experiments that evaluated the trade-off between model performance and computational efficiency. While the loss curves showed continuing minor decrements beyond 10 epochs, we observed that: 1. The rate of improvement in both training and validation loss decreased substantially after epoch 8, with changes in validation accuracy of less than 0.1% per subsequent epoch; 2. The model achieved 98.5% ± 1.48% accuracy across all temporal windows by epoch 10; 3. Extended training beyond 10 epochs (tested up to 20 epochs) produced only marginal improvements (<0.2% increase in accuracy) while significantly increasing computational costs; 4. Early stopping criteria monitoring validation loss showed that the risk of overfitting increased after epoch 10, even though training loss continued to decrease.

**Table 1:** Model’s hyper-parameters

Stage	Hyper-parameter	Value
Image processing	Image size	224 ´ 224
	Sequence length	4
Architecture	ViT	
	Model	vit base patch16 224
	Pretrained	True
	Input channels	3
	Output features	768
	BiLSTM	
	Hidden size	128
	Number of layers	2
	Bidirectional	True
	Output dimension	256
Training	Batch size	16
	Learning rate	1e-5
	Weight decay	0.001
	Optimizer	Adam
	Loss function	CrossEntropyLoss
	Scheduler	StepLR
	Scheduler step size	1
	Scheduler gamma	0.8
	Number of epochs	10
	Dropout	0.5
Data augmentation	Resize	224 ´ 224
	Convert to RGB	Yes
	Normalize mean	0.3796, 0.3915, 0.8996
	Normalize std	0.1860, 0.3054, 0.1428

During training, we employed Adaptive Moment Estimation (Adam) optimizer proposed by Kingma (2015) to update the model parameters. Adam adapts the learning rates for each parameter using estimates of first and second moments of the gradients. Specifically, the learning rate was adjusted using a step learning rate scheduler with a gamma of 0.8 and exponential decay rates for the moment estimates β₁ = 0.9 and β₂ = 0.999. Mixed precision training was enabled through the use of a gradient scaler to enhance computational efficiency. The network architecture, and training parameters are detailed in Table 1.

### Evaluation metrics

The model is trained using CrossEntropyLoss $L\left( {y,\hat y} \right) = - {\rm{ }}\sum\nolimits_1^n {[{y_i}*\log \left( {{{\hat y}_i}} \right)]}$ as the loss function, where $y$ is the true label and $\hat y$ is the predicted probability distribution. In order to comprehensively evaluate the model’s performance, we employed multiple metrics, based on the following basic evaluation components in classification tasks. True Positives (TP): Cases where the model correctly identified the actual physical activity intensity; True Negatives (TN): Cases where the model correctly identified that the activity was not of a particular intensity; False Positives (FP): Cases where the model incorrectly classified an activity as a particular intensity; False Negatives (FN): Cases where the model failed to identify the actual intensity level.

Using these components, we calculate our four key performance metrics, that is,

Accuracy: Measures the overall correct predictions across all intensity levels

$$Acc. = \frac{{Num.\,of\,correct\,predictions}}{{Total\,num.\,of\,predictions}}.$$

Precision: Indicates the model’s ability to correctly identify positive cases for each intensity level

$$Precision = \frac{{TP}}{{TP + FP}}.$$

Recall: Measures the model’s ability to detect all actual positive cases for each intensity level

$$Recall = \frac{{TP}}{{TP + FN}}.$$

Area Under the Receiver Operating Characteristic Curve (AUC-ROC): Evaluates the model’s ability to distinguish between classes across different classification thresholds

$${\rm{AUC}} = \int_0^1 {{\rm{TPR}}\left( {{\rm{FP}}{{\rm{R}}^{ - 1}}\left( {\rm{x}} \right)} \right){\rm{dx,}}}$$

where TPR is the True Positive Rate and FPR is the False Positive Rate

F1 Score: Provides a balanced measure of precision and recall

$$F1{\mkern 1mu} Score = 2 \times {\mkern 1mu} {{Precision \times Recall} \over {Precision + Recall}}.$$

where ${p_o}$ is the observed agreement and ${p_e}$. is the expected agreement by chance.

To visualise the classification performance, confusion matrix is generated for the different activity intensity levels. In these confusion metrics, diagonal elements represent correct predictions where the model’s output matches the true activity intensity and off-diagonal elements indicate misclassifications where the model’s prediction differs from the true intensity.

Additionally, we calculated per-class accuracy to provide detailed insights into the model’s performance for each activity category. These metrics collectively facilitate a comprehensive evaluation of the model’s predictive accuracy and reliability, ensuring robust performance across various levels of physical activity intensity.

### Statistical testing

To assess differences in model accuracy across time windows and physical activity intensities, we employed a two-way analysis of variance (ANOVA), which examines the influence of two independent factors (TW and PAI) and their interaction on a dependent variable (model accuracy). As we hypothesise that the noise levels associated with different intensities and time windows may have a minimal impact on the model’s accuracy, particularly if the model demonstrates high robustness.

The analysis of variance (ANOVA) was conducted to assess differences in model accuracy across physical activity intensities and temporal windows. The following statistical parameters were evaluated: F-value measures the ratio of variance between the groups to the variance within the groups, indicating whether differences between means are significant. Higher F-values suggest greater between-group differences relative to within-group variation. p-value is that statistical significance was set at *p* < 0.05. This threshold was chosen following standard practice in machine learning and physical activity research.

## Results

As illustrated in Fig. 1 overall flowchart shows. First, the process begins with data pre-processing where 3-axis acceleration gravity (time series) data is converted into GAF images using the ENMO method. which effectively captures the temporal correlations of the acceleration data. Second, these GAF images were processed using a ViT to extract meaningful features, that the ViT model segments the GAF images into patches, applies position embeddings, and encodes them using multi-head attention mechanisms to generate robust feature representations.

### Model performance

**Fig. 2:**
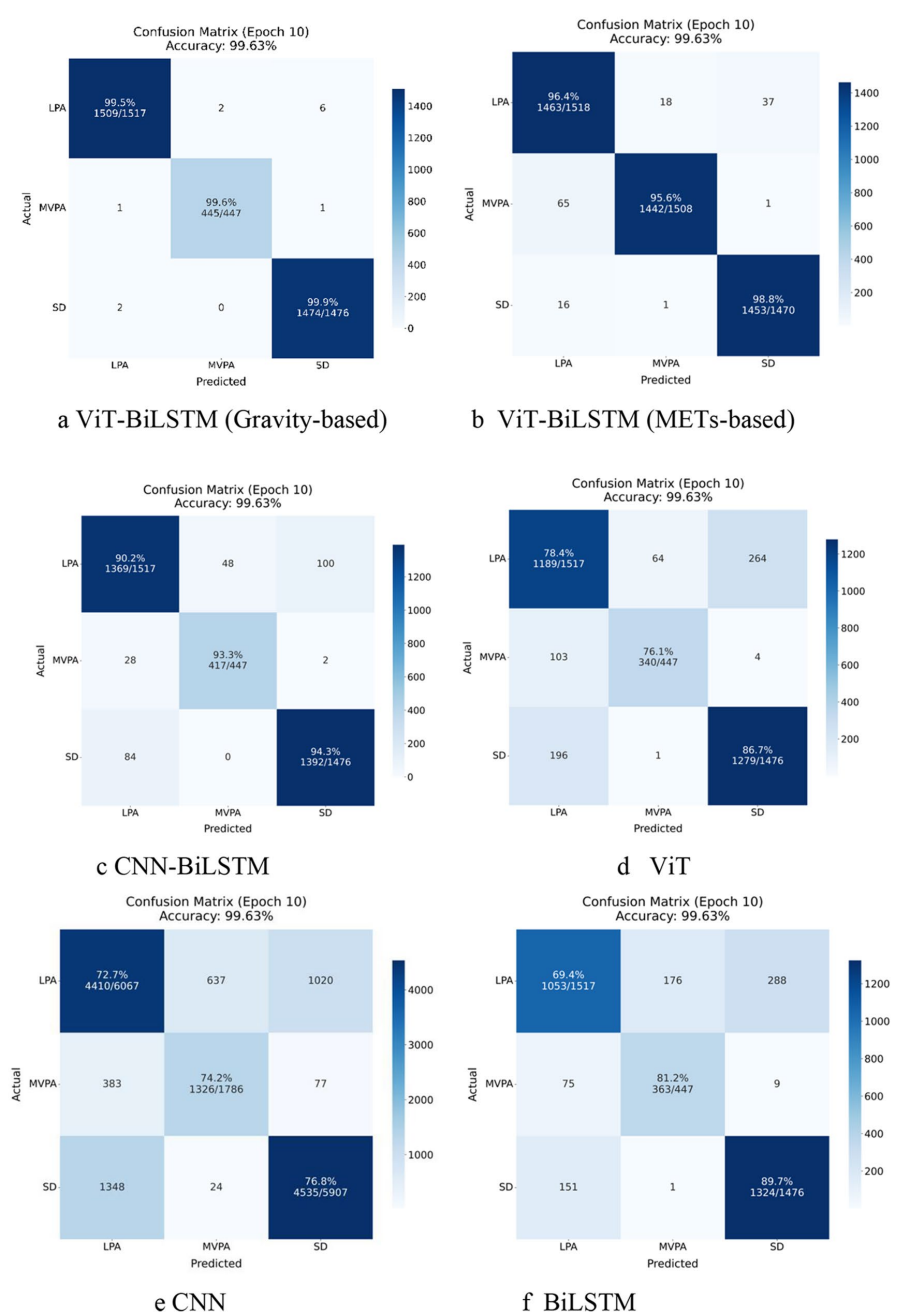
Confusion matrices for comparison of accuracy of different models

Figure 2 compares the confusion matrices for different models: (a) ViT-BiLSTM (Gravity-based), (b) ViT-BiLSTM (METs-based), (c) CNN-BiLSTM, (d) ViT, (e) CNN, and (f) BiLSTM. The proposed ViT-BiLSTM model achieved excellent performance in classifying physical activity intensities compared to others. Specifically, the ViT-BiLSTM model achieved an overall accuracy of 99.63%, with per-class accuracies of 99.5% for LPA, 98.9% for MVPA, and 99.5% for SD. In contrast, the CNN-BiLSTM model reached an accuracy of 92.01%, the ViT model had an accuracy of 80.36%, the CNN model showed an accuracy of 74.57%, and the BiLSTM model attained an accuracy of 80.11%. (The Confusion Matrices 10 epochs for the comparison of different models with 30 TWs as shows in Supplementary material 2.)

**Fig. 3:**
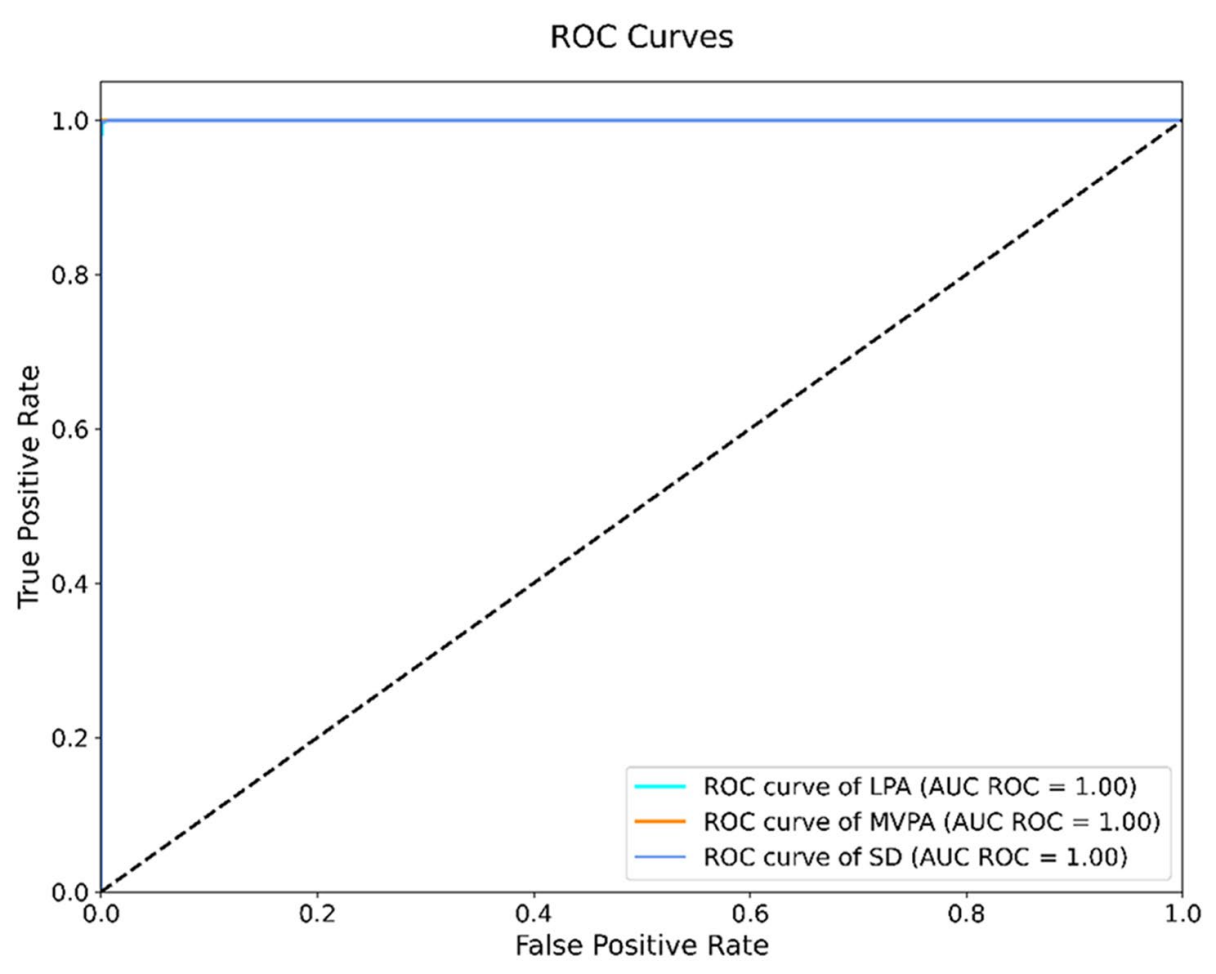
Receiver Operating Characteristic (ROC) curves for different physical activity intensities with TW 15s

The receiver operating characteristic (ROC) curves and their corresponding Area Under the Curve (AUC-ROC) values demonstrate the model’s strong discriminative ability across all activity intensities (Fig. 3). The model achieved excellent discrimination with AUC-ROC values of almost 1.0 for all physical activity intensitives. These high AUC-ROC values indicate the model’s robust capability to distinguish between different activity intensities while maintaining low false positive rates. The ROC curves show particularly strong performance in the low false-positive rate region, suggesting the model maintains high precision even at strict classification thresholds.

**Fig. 4:**
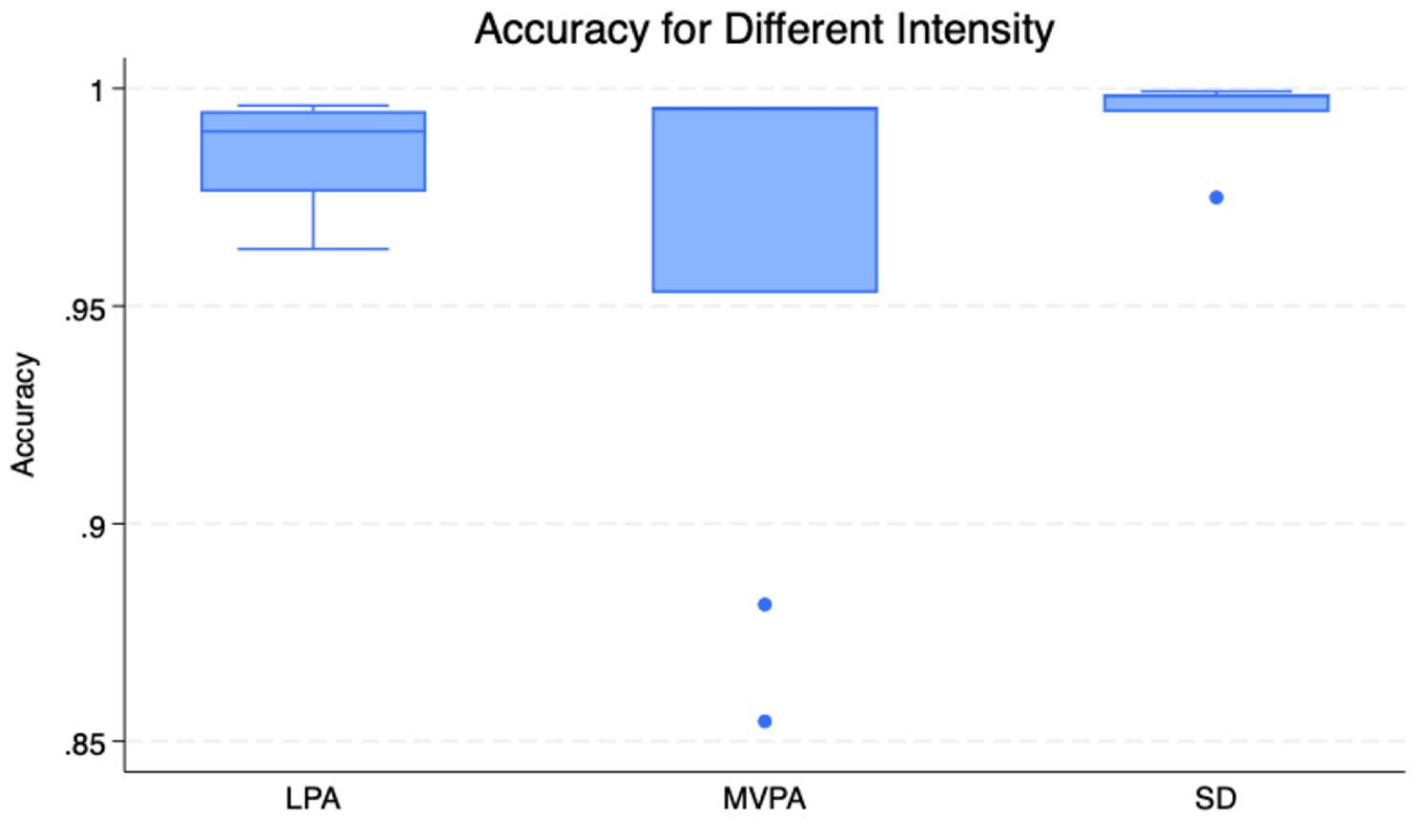
Box plot of model’s accuracy across different pais

**Fig. 5:**
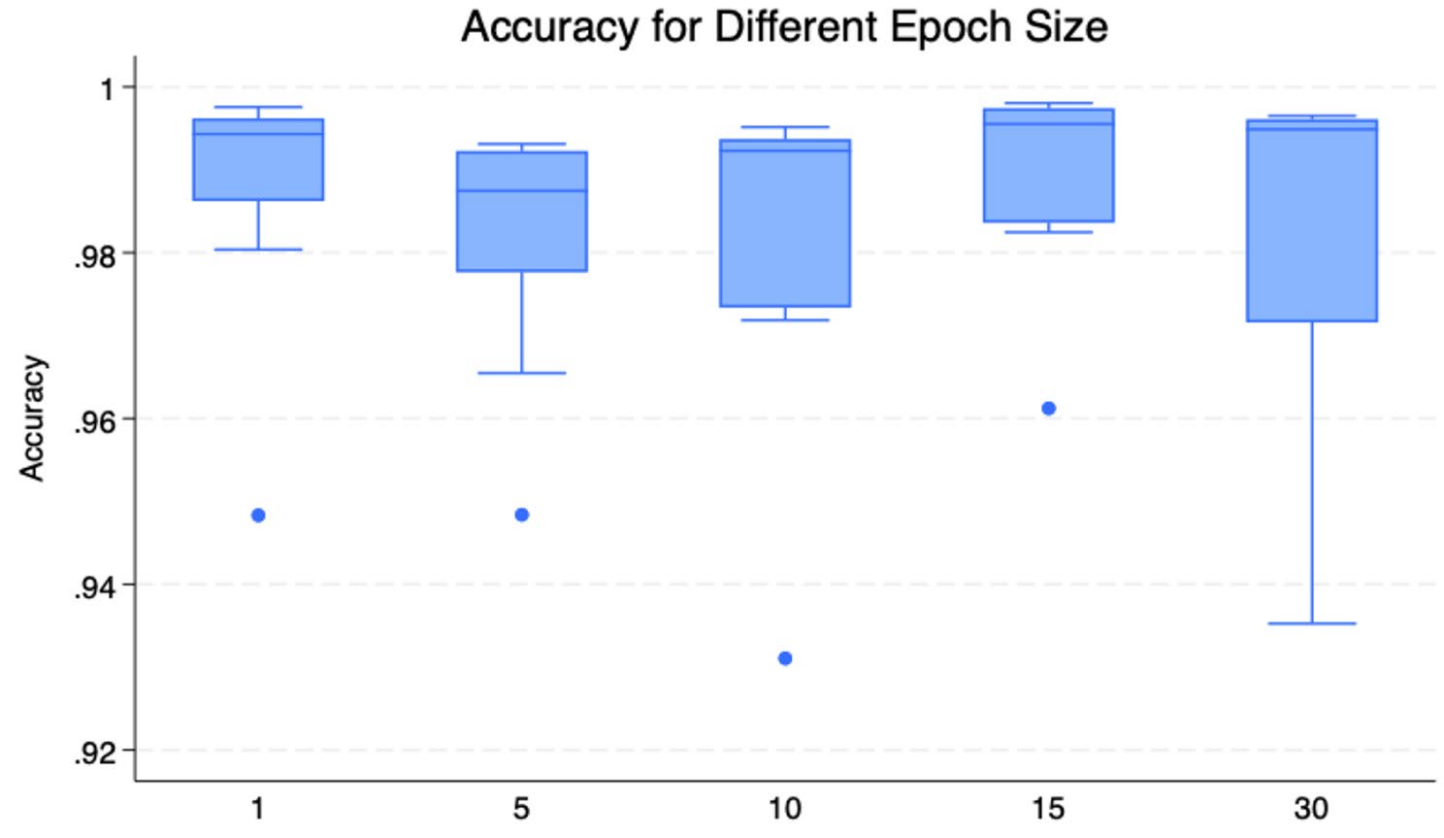
Box plot of model’s accuracy across different temporal window

To evaluate the model’s robustness and reliability, we analysed its performance across different physical activity intensities (PAI) and temporal windows (TW) using both visual and statistical approaches. The distribution and consistency of accuracy scores are visualized through box plots in Figs. 4 and 5, revealing distinct classification patterns across different conditions.

Figure 4 demonstrates the model’s performance across intensity levels (SD, LPA, and MVPA), while Fig. 5 illustrates performance variations across different temporal windows (1s, 5s, 10s, 15s, and 30s). The ANOVA results, presented in Supplementary Material 3, indicate that the model maintains consistent accuracy across both different temporal windows (F = 0.52, *p* = 0.72) and physical activity intensities (F = 2.18, *p* = 0.13), suggesting robust and stable performance regardless of these variations.

Figure 4 shows that the model achieves the highest and most consistent accuracy for SD, with minimal variance and no significant outliers. In contrast, the accuracy for MVPA exhibits a higher variance with notable outliers, indicating less consistency in predictions. LPA demonstrates intermediate performance, with lower variance compared to MVPA but slightly higher than SD.

Figure 5 illustrates the accuracy of the model across different epoch sizes: 1s, 5s, 10s, 15s, and 30s. The whiskers for 5s and 30s are relatively long, indicating greater variability in model predictions for these epoch sizes compared to others. Meanwhile, the average accuracy levels for 15s and 10s are higher, suggesting that the model is most stable when predicting at these epoch sizes. Additionally, the presence of outliers in 1s, 5s, 10s, and 15s may indicate slight inconsistencies in predictions.

**Fig. 6:**
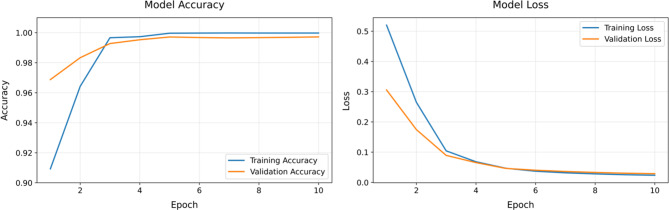
Accuracy and loss curves in the training-validation process

Furthermore, the loss curves were conducted to observe the model’s performance based on the most optimal 15s TW for model predictions. Figure 6 show the results for accuracy and loss curves analysis. The training accuracy (depicted by the orange line) shows a consistent increase from approximately 95% to approximately 100% as the epochs progress from 1 to 10. This indicates that the model is learning well and improving its performance on the training data. The validation accuracy (depicted by the blue line) also shows a steady increase from approximately 96 to 100%, indicating strong generalisation to unseen data. The training loss (depicted by the blue line) decreases sharply from about 9 to nearly 0 as the epochs progress. This rapid decline suggests that the model is quickly learning and minimizing errors on the training data. The validation loss (depicted by the orange line) shows a gradual decrease from around 1 to near 0, indicating a steady improvement in model performance on the validation data. The relatively small and stable loss values demonstrate that the model is not overfitting. These curves show that the model performs well during training and maintains good generalisation on validation data, as indicated by the close alignment between training and validation accuracy and the decreasing loss values. The model’s consistent performance across epochs highlights its robustness and effectiveness in learning the underlying patterns in the data. Supplementary materials 4 shows accuracy and loss Curves for different TWs, which are also stability across epochs.

Finally, from the results in Table 2, it can be observed how different intensities and TWs affect the model’s accuracy. The model’s performance varies with different TWs used for generating images. Specifically, the 15s TW shows the best performance, achieving the highest train (0.99 ± 0.01) and test (0.99 ± 0.01) accuracies. In contrast, the 5s TW exhibits the lowest train (0.981 ± 0.01) and test (0.981 ± 0.01) accuracies. When considering different PAIs across various TWs, the model consistently achieves higher accuracy in predicting SD (0.989 ± 0.01) compared to LPA (0.982 ± 0.02) and MVPA (0.982 ± 0.03). This trend is apparent across all TWs, indicating that SD is easier for the model to predict accurately. In shorter epochs like 1s or 5s, the model shows better prediction accuracy for MVPA (0.992 ± 0.007; 0.985 ± 0.01) compared to other intensities. However, in longer epochs such as 30s, the model achieves the lowest prediction accuracy for MVPA (0.965 ± 0.05), but higher for LPA (0.984 ± 0.01), and highest for SD (0.995 ± 0.007). The 1s and 15s epochs demonstrated markedly higher standard deviations in test loss (SD = 0.20 and SD = 0.21, respectively) compared to other temporal windows (5s: SD = 0.007; 10s: SD = 0.006; 30s: SD = 0.006). Similarly, training loss values showed substantial variation, with the 1s and 15s epochs exhibiting higher standard deviations (SD = 2.8 and SD = 2.91) compared to other epochs (5s: SD = 0.19; 10s: SD = 0.18; 30s: SD = 0.17). This pattern suggests that while these temporal windows achieved high accuracy (1s: 0.987 ± 1.5; 15s: 0.99 ± 0.01), they also experienced greater fluctuations in model performance during training. The increased variability might be attributed to two-folds: (1) the challenge of capturing complete activity patterns in very short time segments (1s), leading to more unstable predictions; (2) add the explanation for 15s time window. These findings highlight an important trade-off between prediction accuracy and stability across different temporal windows, suggesting that window selection should consider both performance metrics and consistency requirements for specific applications.

**Table 2:** Summary of model performance across different TWs and physical activity intensities

Epoch (training times)	Train accuracy	Test accuracy	Train loss	Test loss
	Mean (SD)	Mean (SD)	Mean (SD)	Mean (SD)
Total (*n* = 50)	0.984 (0.17)	0.985 (1.48)	0.096 (0.2)	0.088 (0.16)
LPA		0.982 (0.02)		
MVPA		0.982 (0.02)		
SD		0.989 (0.01)		
1s epoch (*n* = 10)	0.987 (0.01)	0.987 (1.5)	0.2 (0.28)	0.194 (0.20)
LPA		0.981 (0.03)		
MVPA		0.992 (0.007)		
SD		0.990 (0.11)		
5s epoch (*n* = 10)	0.981 (0.01)	0.981 (0.01)	0.014 (0.019)	0.007 (0.007)
LPA		0.977 (0.02)		
MVPA		0.985 (0.01)		
SD		0.982 (0.01)		
10s epoch (*n* = 10)	0.982 (0.02)	0.982 (0.02)	0.0131 (0.018)	0.006 (0.006)
LPA		0.978 (0.03)		
MVPA		0.981 (0.03)		
SD		0.988 (0.01)		
15s epoch (*n* = 10)	0.99 (0.01)	0.99 (0.01)	0.23 (0.29)	0.228 (0.21)
LPA		0.99 (0.006)		
MVPA		0.987 (0.02)		
SD		0.992 (0.009)		
30s epoch (*n* = 10)	0.981 (0.02)	0.986 (1.39)	0.014 (0.017)	0.007 (0.006)
LPA		0.984 (0.01)		
MVPA		0.965 (0.05)		
SD		0.995 (0.007)		

## Discussion

This study innovatively combines ViT and BiLSTM to predict PAI using gravity-based acceleration to generate images. It is also the first to consider the impact of different intensities and TWs on model robustness. Additionally, this study investigates how PAI and TW impact the accuracy of the ViT-BiLSTM model. The present study demonstrates that: (1) the ViT-BiLSTM model exhibits high performance in predicting PAI; (2) the study confirms the feasibility of using gravity-based acceleration for intensity classification tasks. The gravity-based calculation of PAI significantly enhances model accuracy compared to traditional MET-based methods; (3) the model’s high performance reveals good robustness and reliability, unaffected by variations in intensity and TWs; and the model consistently improves its performance across epochs, with both training and validation accuracy increasing to near 100%, and training and validation loss decreasing to nearly zero in accuracy and loss curve analyses. (4) the TW and PAI are the potential factors that contribute to the model’s accuracy.

The present study applied the hybrid model of ViT-BiLSTM in classifying LPA, MVPA, SD achieving an overall accuracy exceeding 99.6% (gravity-based) and 96.9% (METs-based) using a 30-s TW, which is higher than other previous models [9, 15–17, 19, 21, 32, 33]. The present results are directly comparable with those reported in 8 published studies. Three of these studies used traditional machine learning methods on the same dataset (Capture-24) we used, obtaining overall accuracies of 80% [20], 88% [33] and 87% [32]. This may be due to traditional machine learning models relying on linear regression, which have limitations in predicting the complex variations in intensity during PA. The other five studies were based on deep learning methods, with four of them using laboratory-collected accelerometer data. In these five studies, one study applied ANN and k–NN models, resulting in 92% and 80% accuracy, respectively [19]. Two studies used CNN models: one study with data from five accelerometers showed a range of 92%–98% accuracy [21]; while another study achieved accuracies of 63%, 84.2%, and 85.4% for MVPA, LPA, and SD, respectively [16], Additionally, a study implementing a BiLSTM model with data from three accelerometers achieved 90% accuracy [9]. Considering only a single model framework and a single data dimension may limit the model’s ability to accurately assess PAI, as ANN, k–NN, and CNNs alone may not handle time series data effectively, Moreover, BiLSTM models without a preceding convolutional layer cannot extract spatial features.

Furthermore, compared to a recent study by Farrahi, Muhammad [15], which used the AccNet24 model and achieved accuracies of 98.6% for SD, 95.6% for LPA, and 94.7% for MVPA using METs-based PAI on the Capture-24 dataset with a 30-s TW window, our ViT-BiLSTM model, also based on METs for intensity classification and using the same dataset and TW, achieved higher accuracies of 98.2% for SD, 96% for LPA, and 96.3% for MVPA. This may because the ViT-BiLSTM model may provide the global information and utilises attention mechanisms, excelling at capturing the magnitude of intensity under variable visual conditions with the ViT model, while the integration with the BiLSTM model enhances its ability to accurately capture dynamic physical activities. Additionally, when our ViT-BiLSTM model, based on gravity-based acceleration for intensity classification and using the same dataset and TW, achieved higher accuracies of 99.9% for SD, 99.5% for LPA, and 99.6% for MVPA, this may confirm the feasibility of using gravity-based acceleration for intensity classification tasks. The gravity-based calculation of PAI enhances model accuracy compared to traditional MET-based methods. This may be because the present study used the gravity-based acceleration to classify images for model training. Gravity-based acceleration, to some extent, surpasses the METs-based method, reducing the likelihood of misclassification for PAI during image generation.

Meanwhile, the ViT-BiLSTM model demonstrated excellent robustness and generalisation. ANOVA showed no accuracy variation across PAIs (F = 2.18, *p* = 0.13) and TWs (F = 0.52, *p* = 0.72). The ViT-BiLSTM model consistently improved its performance across epochs, with both training and validation accuracy increasing to nearly 100%, and training and validation loss decreasing to nearly zero in accuracy and loss curves analysis. Again, the present study observed that different models exhibited similar trends when predicting PAI, SD is relatively easier to predict, likely due to the stable nature of sedentary behaviour. In contrast, the prediction accuracy for LPA and MVPA is more likely lower than for SD, confirming the complexity of LPA and MVPA behaviours [15, 21], However, the ViT-BiLSTM model can almost overcome the variations between behaviours, achieving nearly perfect accuracy in predicting different PAIs. The present study tested the variation in the model’s accuracy across different PAIs, The ViT-BiLSTM model results for predicting SD, LPA, and MVPA were 99.9%, 99.5%, and 99.6%, respectively.

PA characteristics may be a potential factor affecting the performance of the model and should be taken into consideration. Previous studies have also reported similar findings. Nawaratne, Alahakoon [16] found that SB achieved correct predictions of 85.4%, LPA achieved correct predictions of 84.2%, and MVPA achieved correct predictions of 63.1% based on a 60-s TW. In contrast, Widianto, Sugiarto [21] showed accuracies of 98%, 95%, and 97% for SD, LPA, and MVPA, respectively, based on a 1-s TW. Also, Recent studies have reported similar findings, showing that a 1-s window performs best in predicting any intensity level. This is mainly because a 1-s window calculates acceleration more accurately, reducing the likelihood of values being averaged out in images. Additionally, for predicting LPA and SD, longer windows may provide higher accuracy, while shorter windows are more effective in predicting MVPA compared to LPA, as MVPA usually involves a amount of activity expenditure over a short period [34], Consequently, MVPA often lasts only a few seconds in adults, especially middle-aged or older individuals than LPA and SD [35]. However, using longer windows may introduce noise to the model due to greater variations in MVPA magnitude, which can affect the model’s accuracy during data preprocessing. Although the impact of this factor on the model in this study was very slight, considering the characteristics of adult PA behaviour may help overcome some noise effects in future model development in this area. Additionally, Outliers mainly refer to the lowest values observed during the first epoch of the model, with a small difference of approximately 2–5% compared to the average accuracy, which indirectly demonstrates the good convergence performance of the model.

Strength of the present study demonstrates several notable strengths in its approach to PAI classification. It innovatively combines ViT and BiLSTM models, fully leveraging the advantages of both architectures to enhance classification accuracy. The use of gravity-based acceleration methods for classifying PAI marks a significant improvement over traditional METs-based approaches, potentially reducing classification errors. This study considers different TWs and PAIs, providing a nuanced understanding of how these factors impact model performance. By utilising the real-world Capture-24 dataset, the study enhances the practical applicability of its findings. Furthermore, the rigorous evaluation of model performance and stability through multiple methods, including ANOVA, confusion matrices, and accuracy and loss curves, underscores the study’s methodological robustness. These strengths collectively contribute to the study’s advancement in the field of PA monitoring and classification using deep learning techniques. In this study, the ViT-BiLSTM model demonstrated high accuracy in classifying physical activity intensities, Future efforts could focus on optimizing the model for such environments by leveraging techniques like model pruning, which reduces unnecessary parameters, and quantization, which decreases the precision of weights to lower memory usage and computational demands. Additionally, lightweight architectures could be explored as alternatives to the current design. These strategies would maintain model performance while enabling its application in real-world scenarios. A limitation of the present study is that it focused solely on adult populations and used a single intensity threshold to calculate activity intensity. Future work should include multiple threshold comparisons to evaluate the model’s robustness under diverse conditions.

## Conclusion

The ViT-BiLSTM model proposed in this study demonstrated exceptional performance in classifying PAI using gravity-based acceleration data, achieving an overall accuracy of 99.63%. The model exhibited excellent robustness and reliability across different TWs and activity intensities. The research found that a 15-s TW yielded the best performance in most cases, and the model’s accuracy in predicting sedentary behaviour was slightly higher than for light and moderate-to-vigorous activities. These findings provide important methodological references for future PA monitoring and classification research. Future study should focus on validating these findings across additional datasets using different PA thresholds and exploring the model’s performance with a broader range of physical activities to further enhance its practical applications.

## Electronic supplementary material

Below is the link to the electronic supplementary material.

Supplementary Material 1

Supplementary Material 2

Supplementary Material 3

Supplementary Material 4

Supplementary Material 5

## Abbreviations

ViT: Vision Transformer
BiLSTM: Bidirectional Long Short-Term Memory
PAI: Physical Activity Intensity
TW: Temporal Window
CNN: Convolutional Neural Network
RNN: Recurrent Neural Network
ENMO: Euclidean Norm Minus One
GAF: Gramian Angular Field
LPA: Light Physical Activity
MVPA: Moderate-to-Vigorous Physical Activity
SB: Sedentary Behaviour
METs: Metabolic Equivalents
ANOVA: Analysis of Variance
