# Supplementary material for: A novel ViT-BILSTM model for physical activity intensity classification in adults using gravity-based acceleration
Source: BMC Biomed Eng. 2025 Feb 1;7:2. doi: 10.1186/s42490-025-00088-2 (PMC11786420; doi:10.1186/s42490-025-00088-2)

This supplementary material shows the definitions of the x, y, and z axes of Axivity AX3 accelerometer, as well as x, y, and z axes in different physical activity intensity.


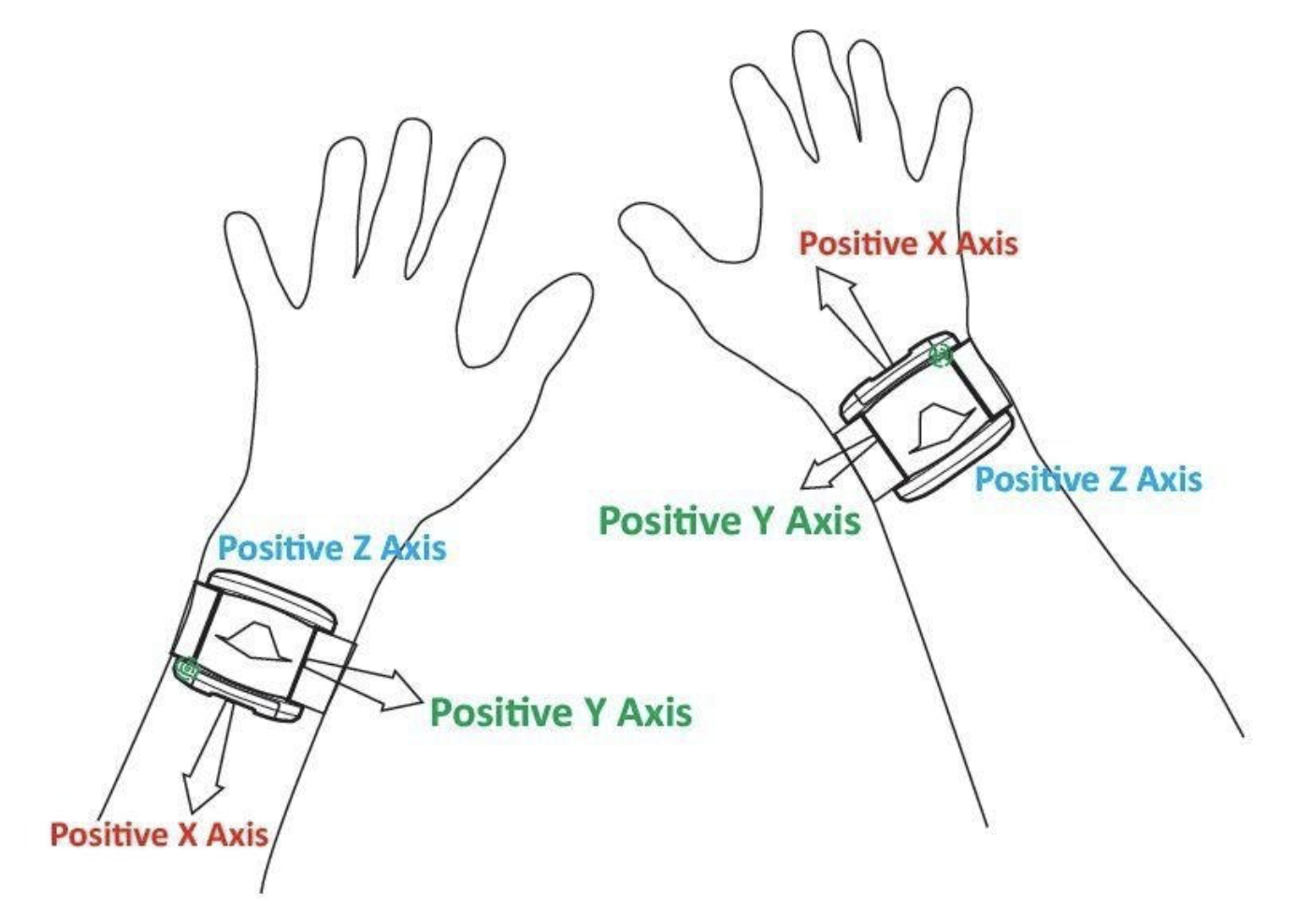


Sensors on participants' wrists (adapted from Axivity AX3 User Guide(2013b))

The diagram shows the placement and orientation of the Axivity AX3 accelerometer on the wrists of both hands. The three axes of the accelerometer are defined in a three-dimensional coordinate system: the x-axis points in the front-back direction along the wrist, the y-axis points in the side-to-side direction (perpendicular to the arm), and the z-axis points upward, perpendicular to the surface of the device (toward the hand).

Light physical activity


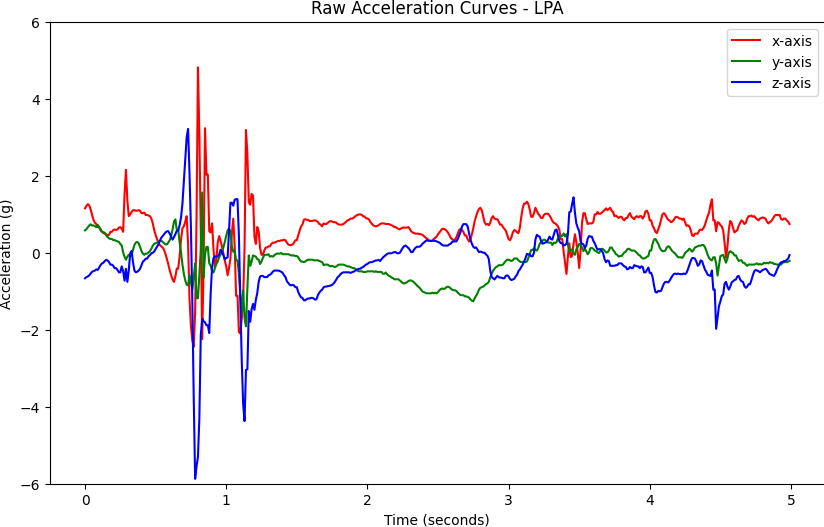


Moderate-to-vigorous physical activity


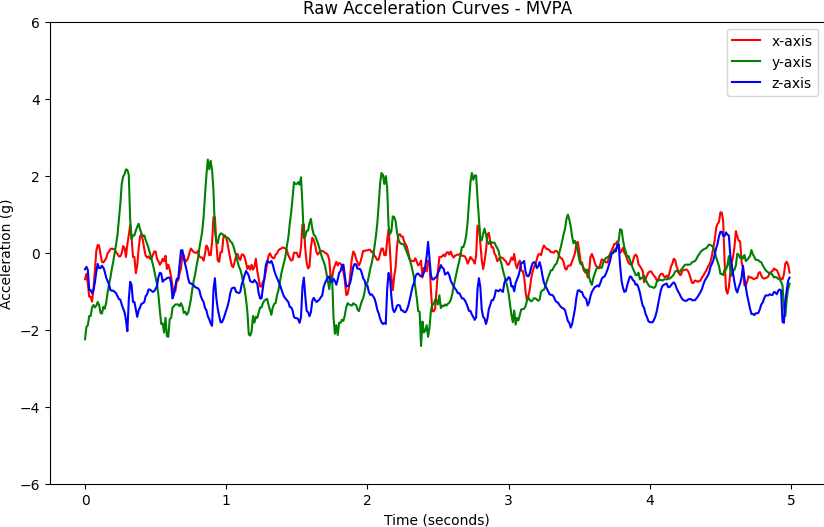


Sedentary


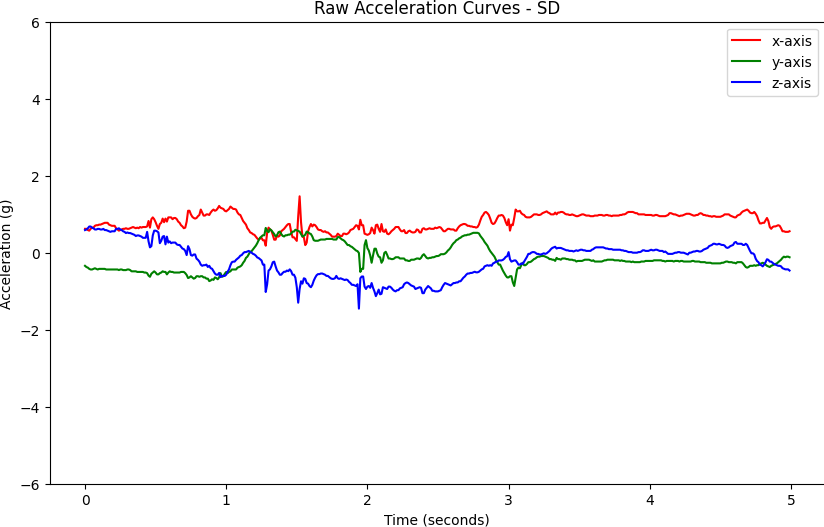

Supplement: Supplementary file 1 — Supplementary Material 1 [file 42490_2025_88_MOESM1_ESM.docx]
