# Supplementary material for: A novel ViT-BILSTM model for physical activity intensity classification in adults using gravity-based acceleration
Source: BMC Biomed Eng. 2025 Feb 1;7:2. doi: 10.1186/s42490-025-00088-2 (PMC11786420; doi:10.1186/s42490-025-00088-2)

10 epochs for the comparison of different models with 30 TWs

Confusion Matrices for Different Models

This figure presents the confusion matrices comparing the performance of different models in classifying physical activity intensities over 10 epochs with a 30-second temporal window.

**1 Vit Model (gravity-based for encoding)**


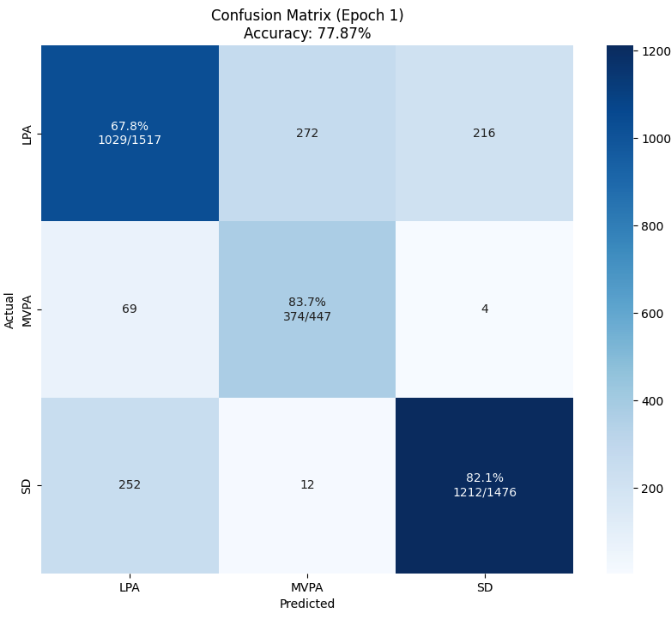


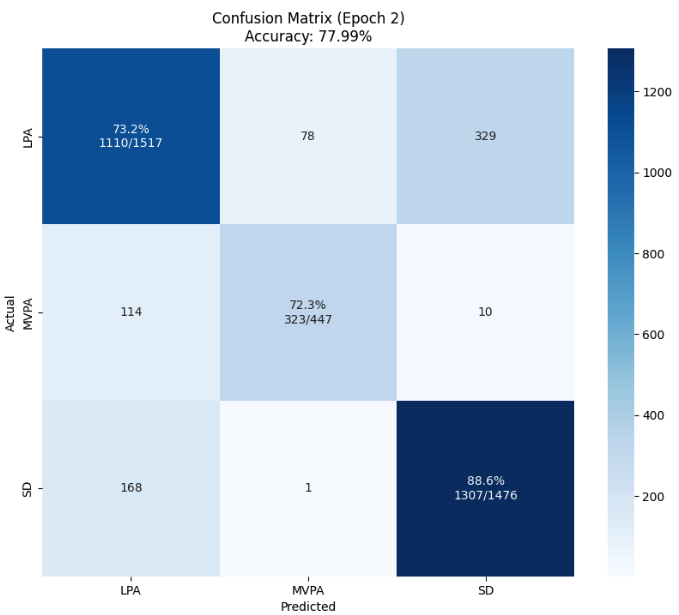


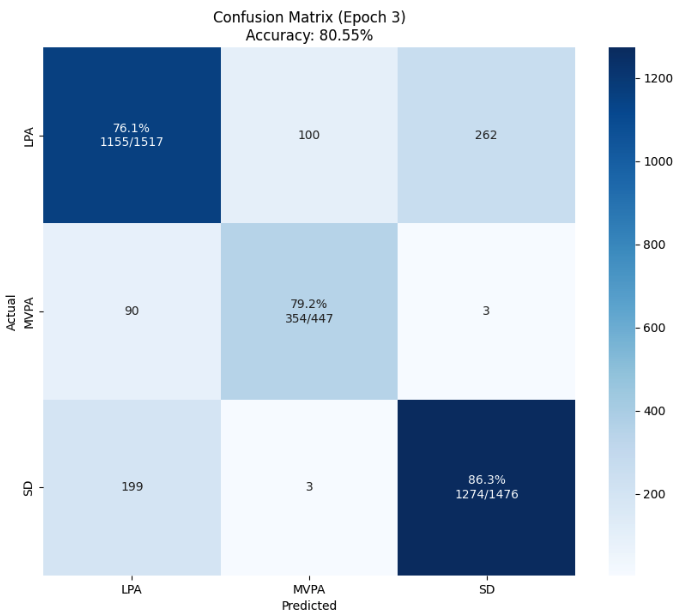


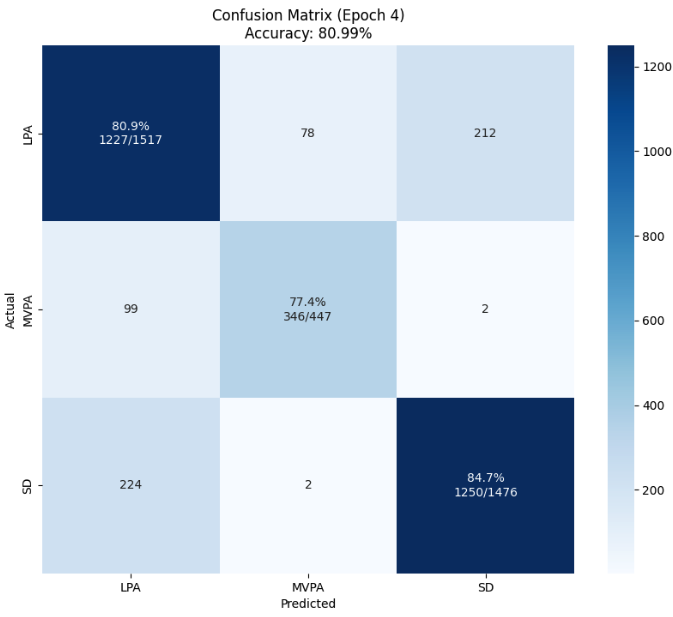


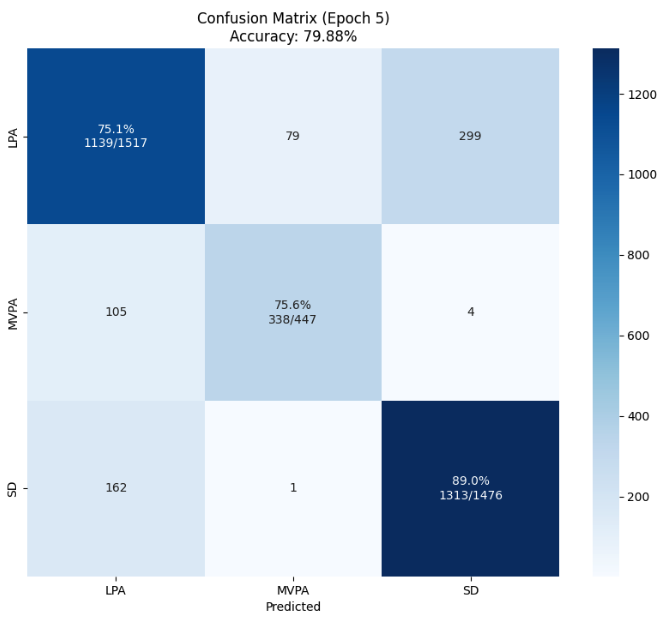


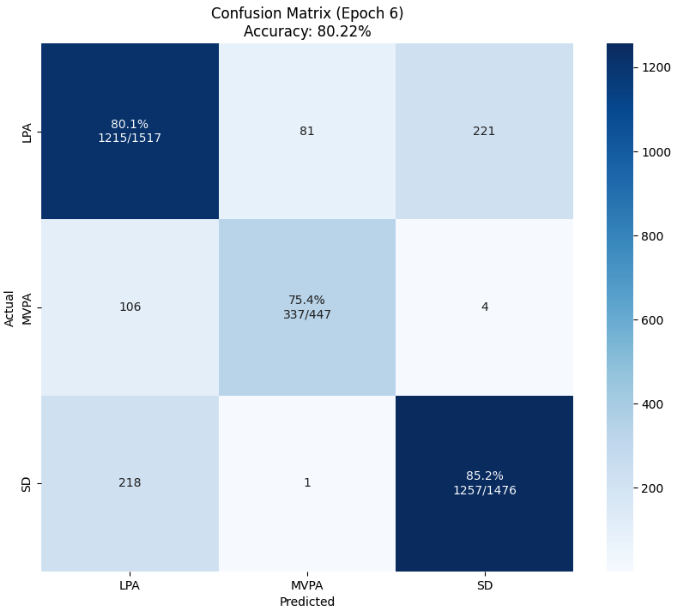


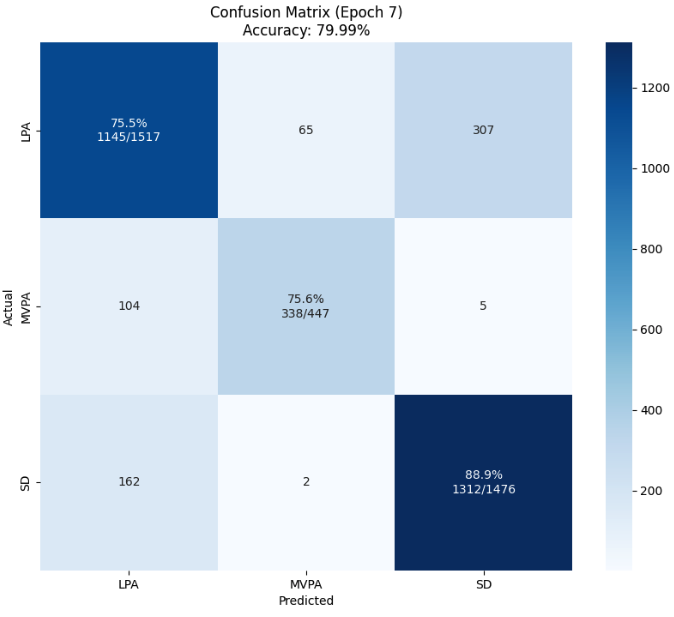


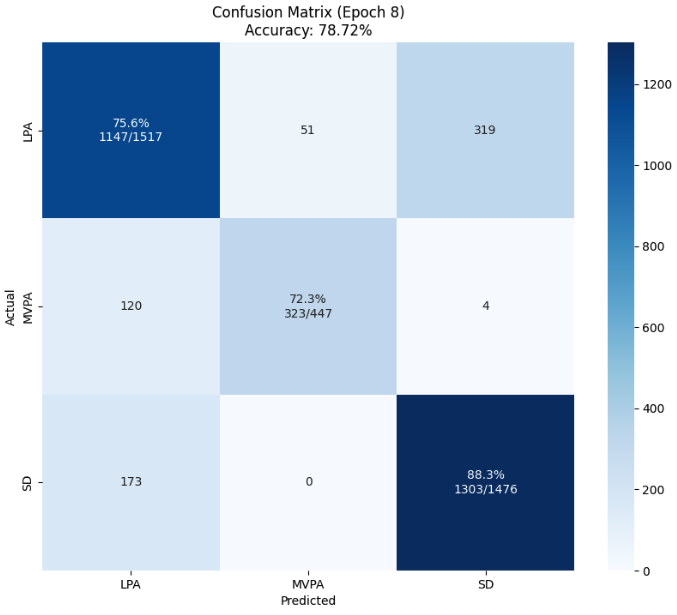


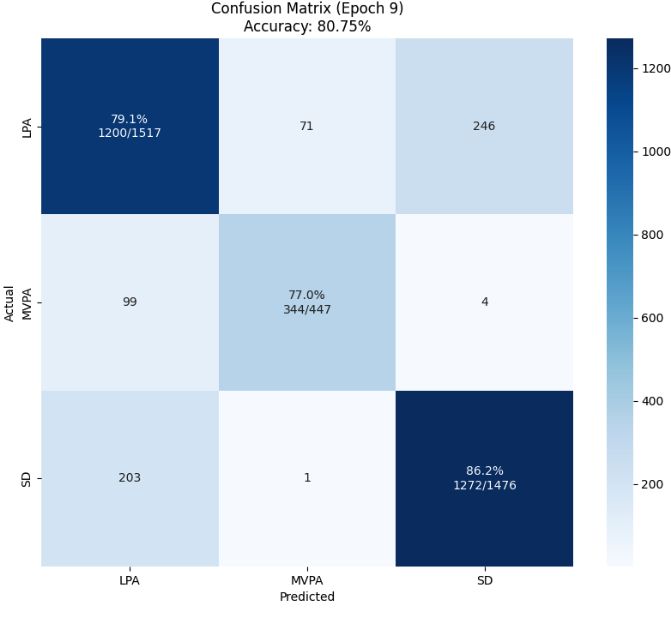


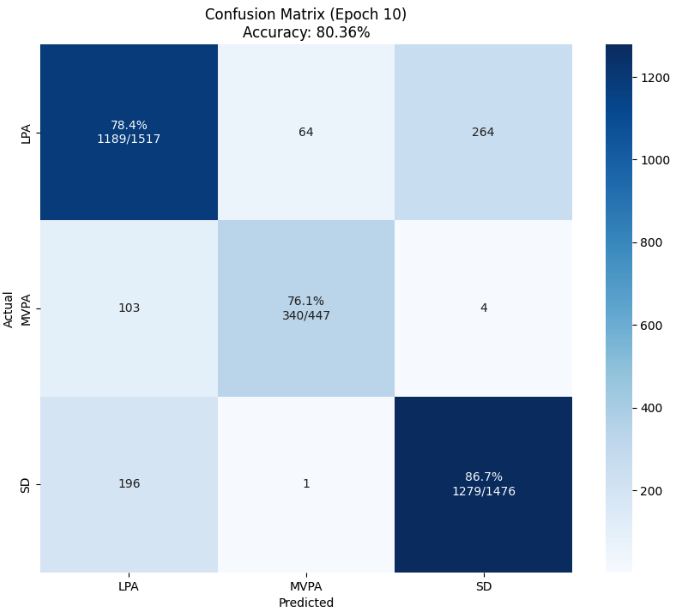


**2 BiLSTM (gravity-based for encoding)**


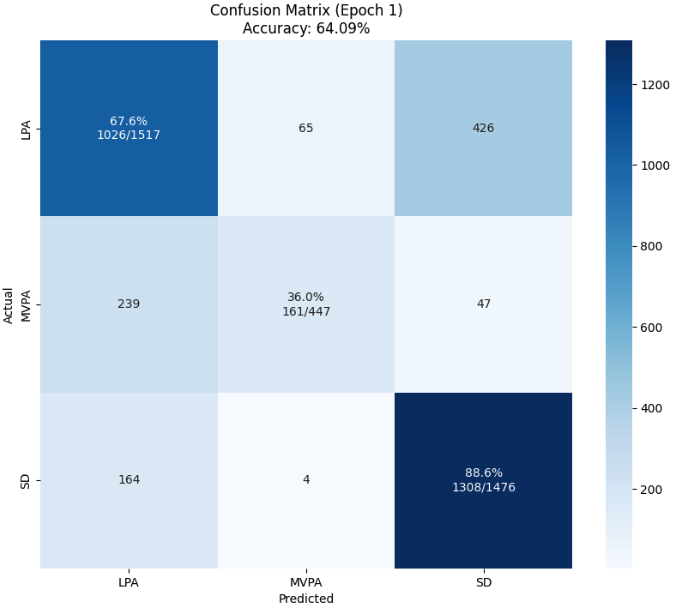


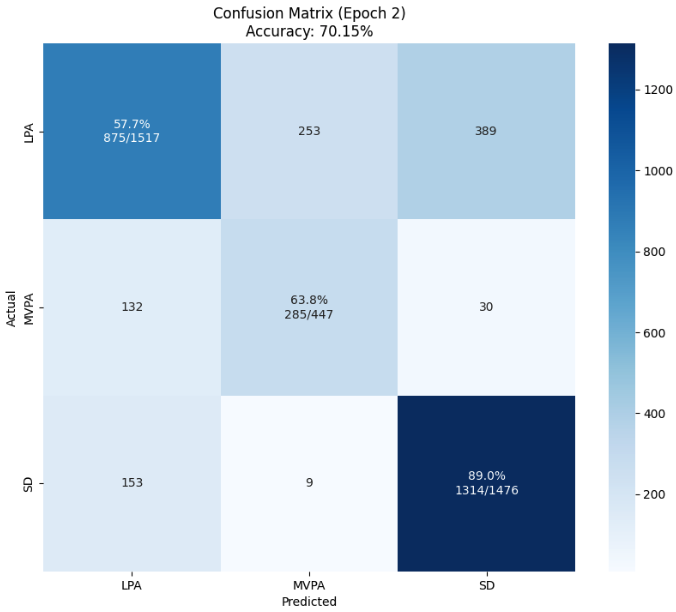


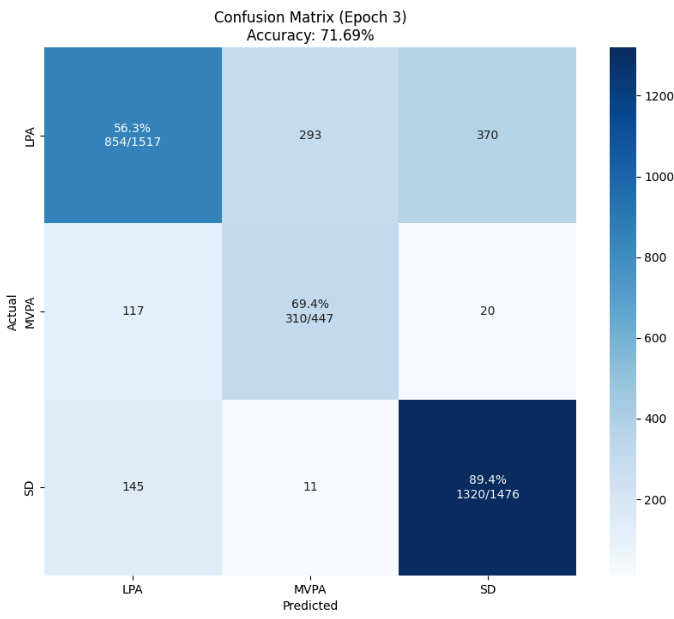


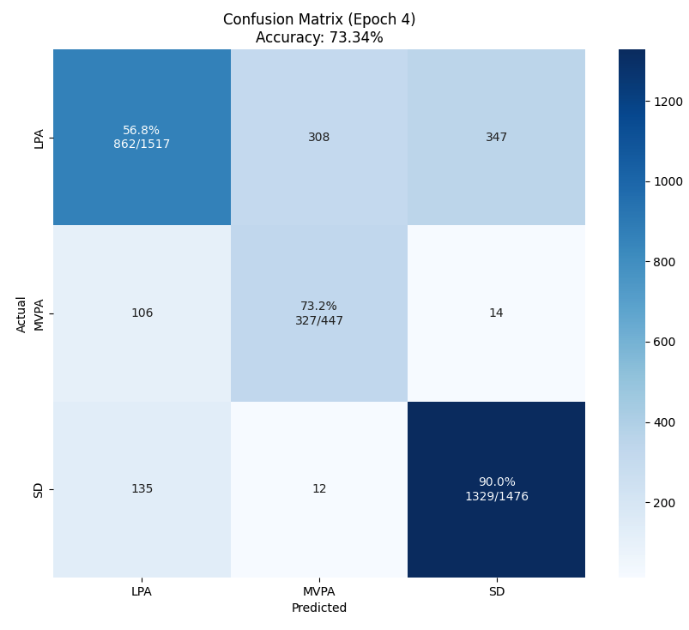


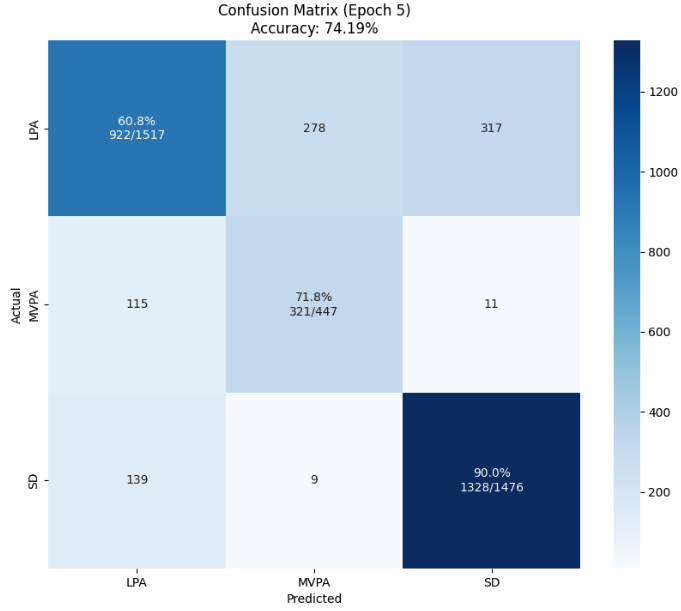


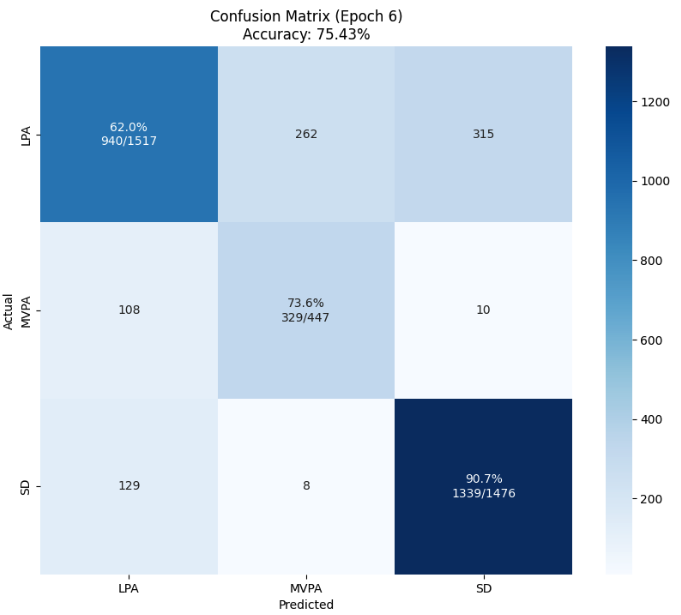


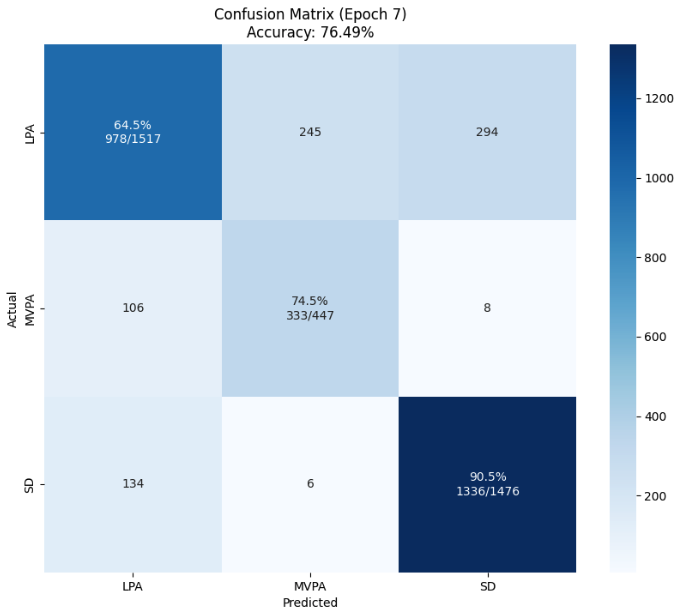


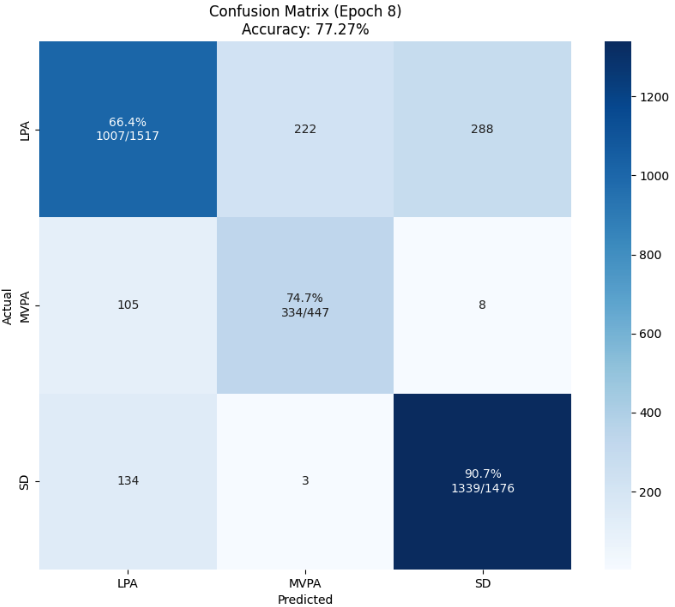


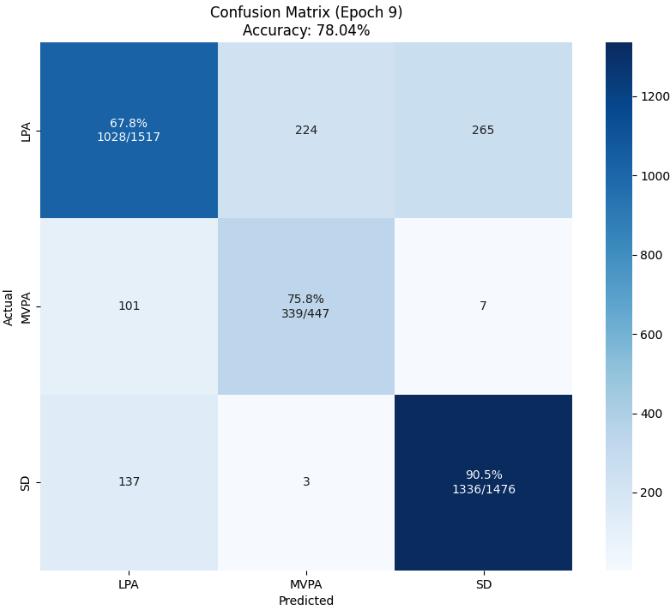


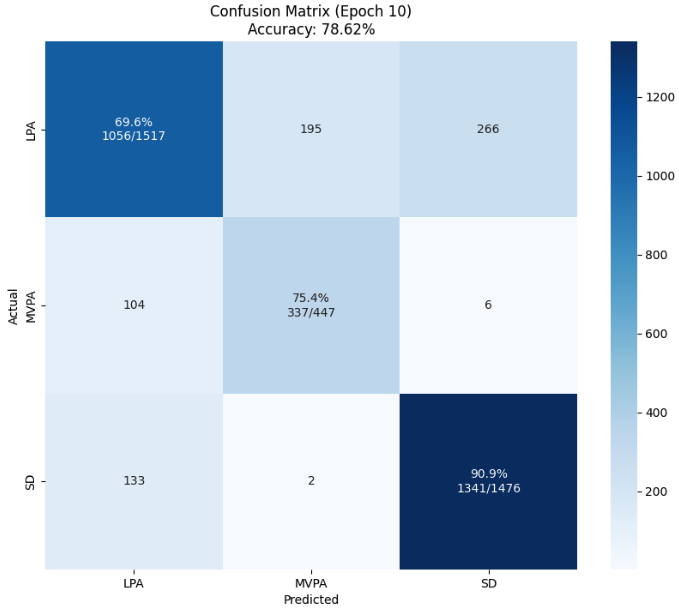


**3 CNN Model (gravity-based for encoding)**


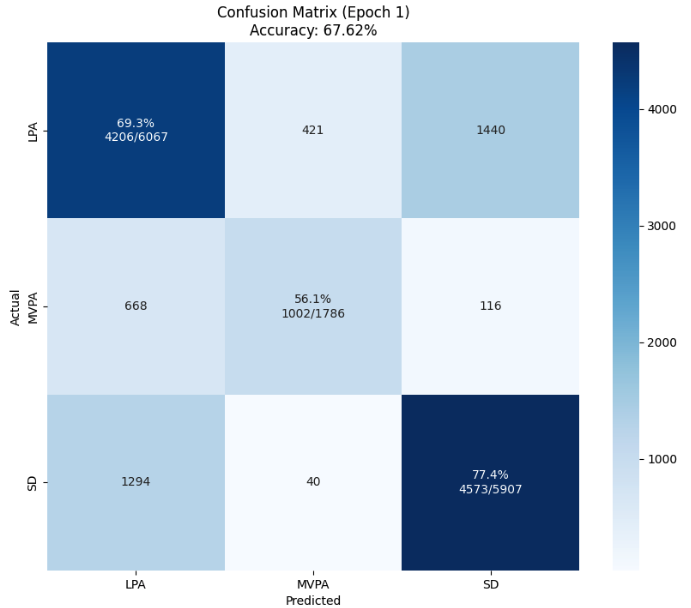


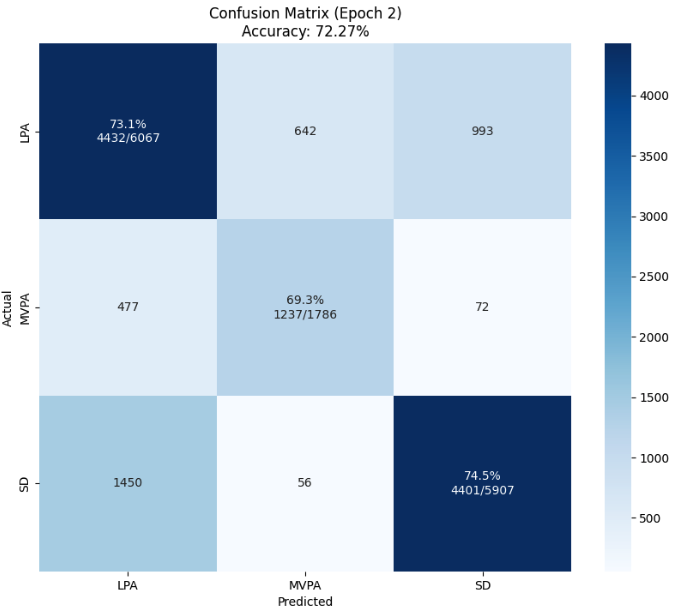


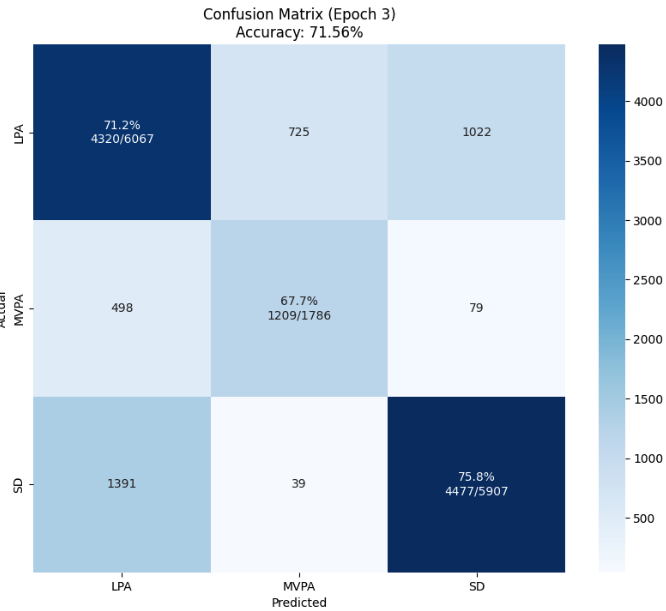


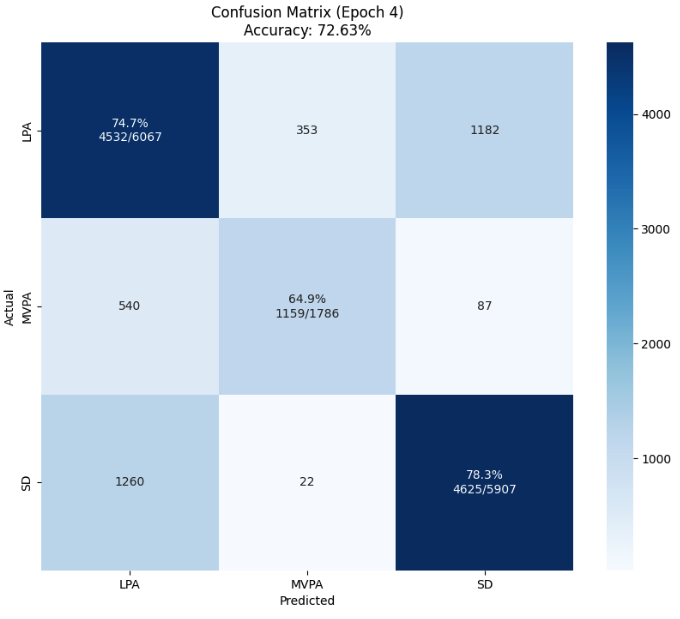


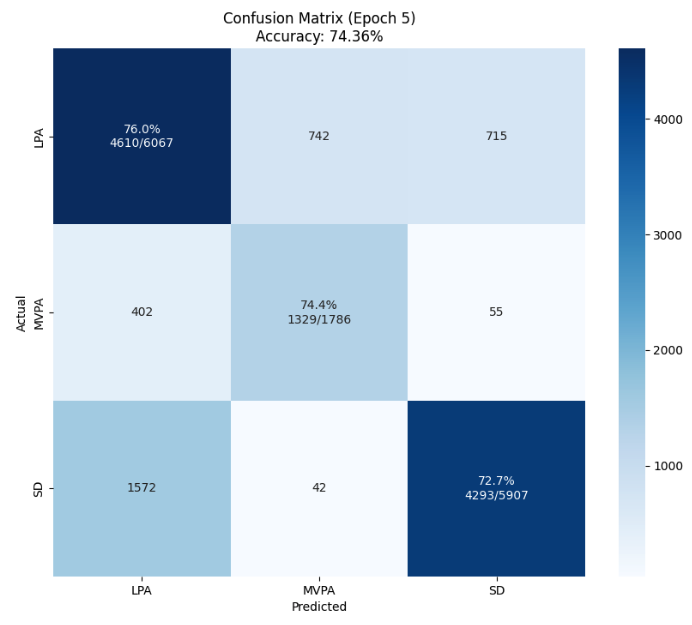


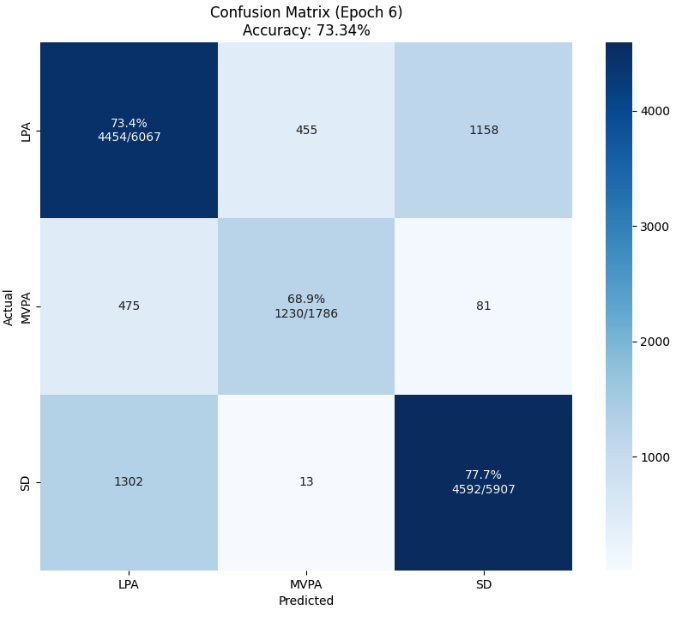


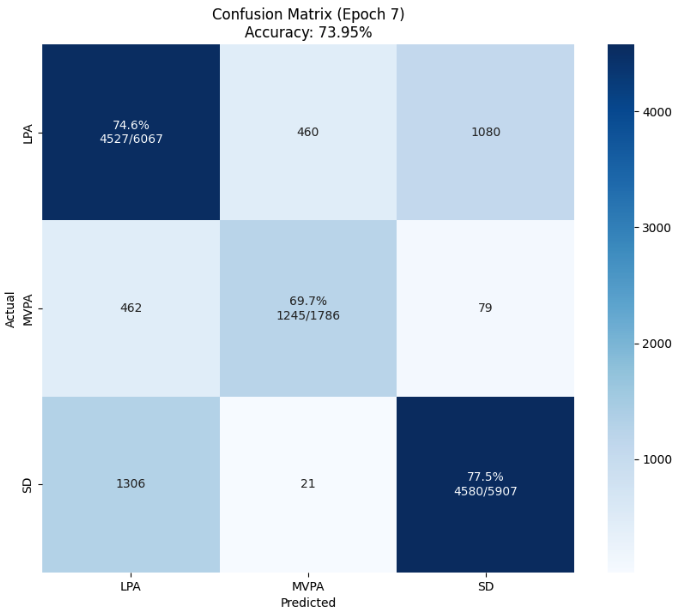


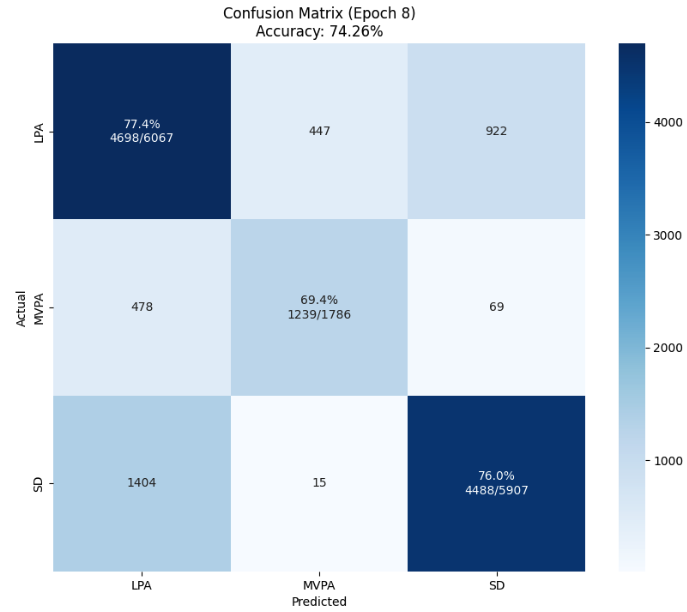


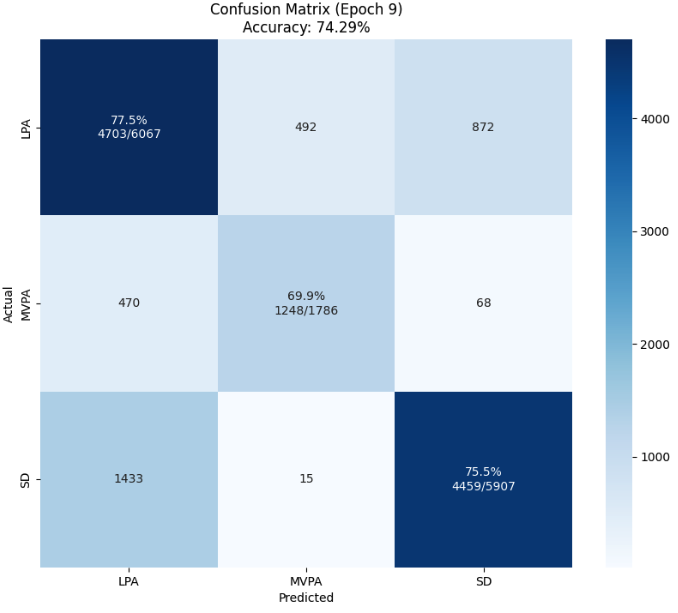


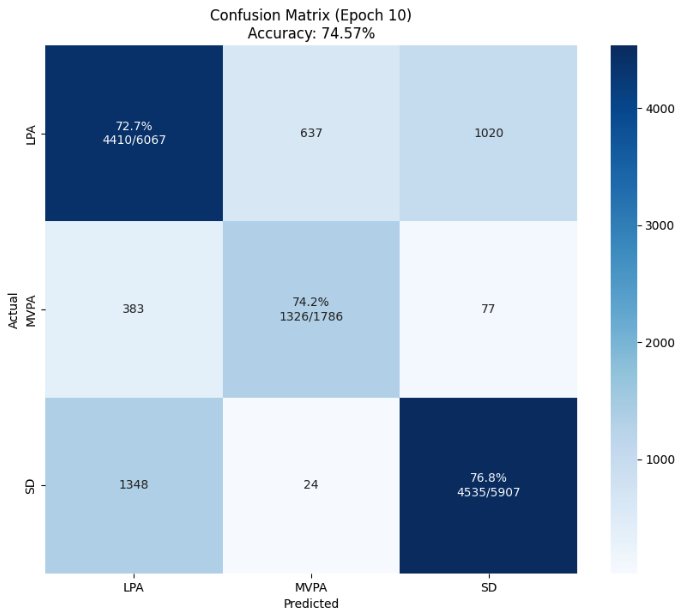


**4 CNN_BiLSTM (gravity-based for encoding)**


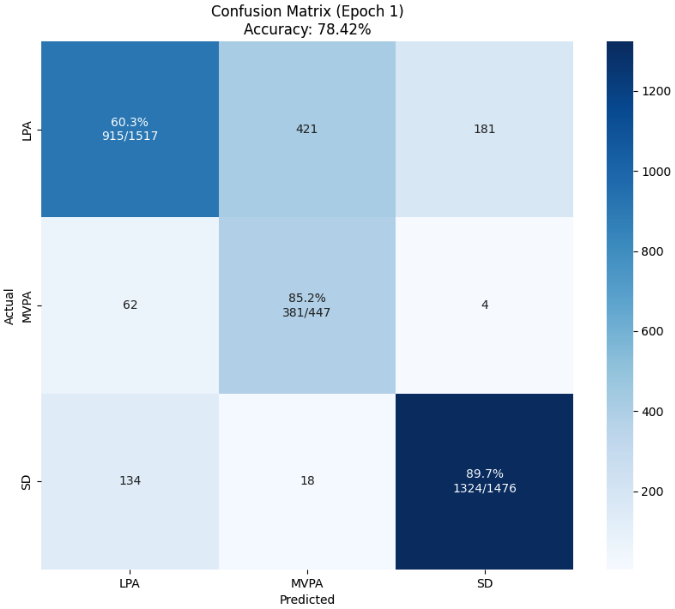


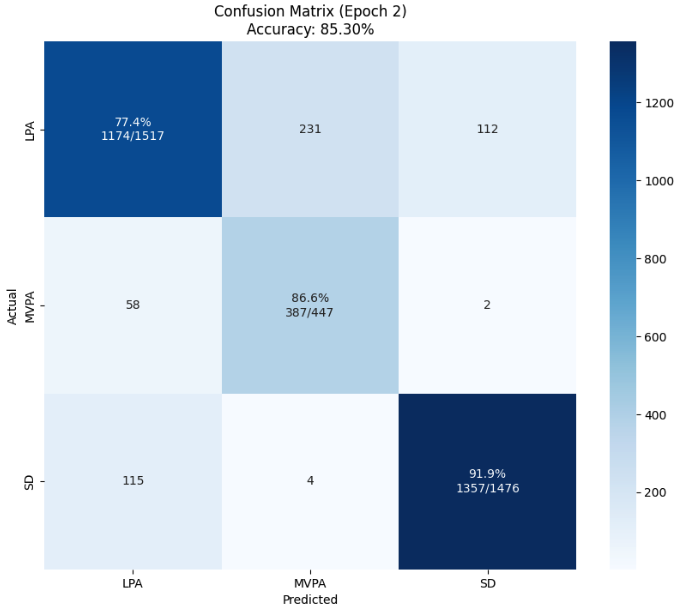


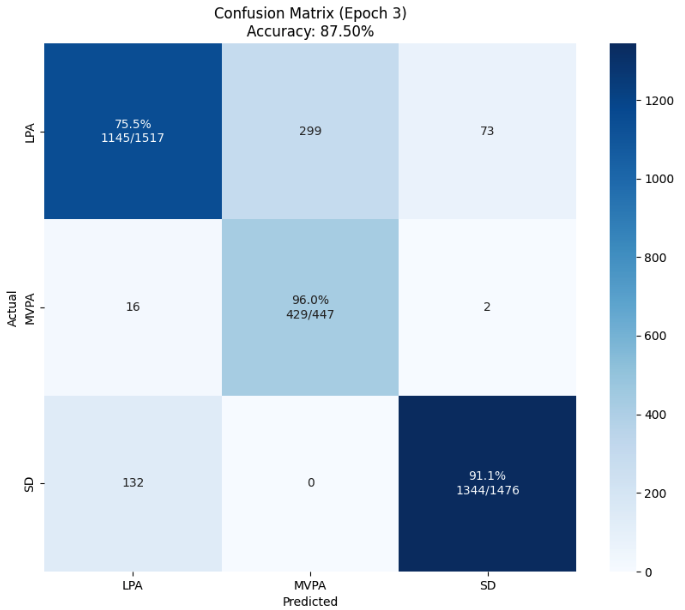


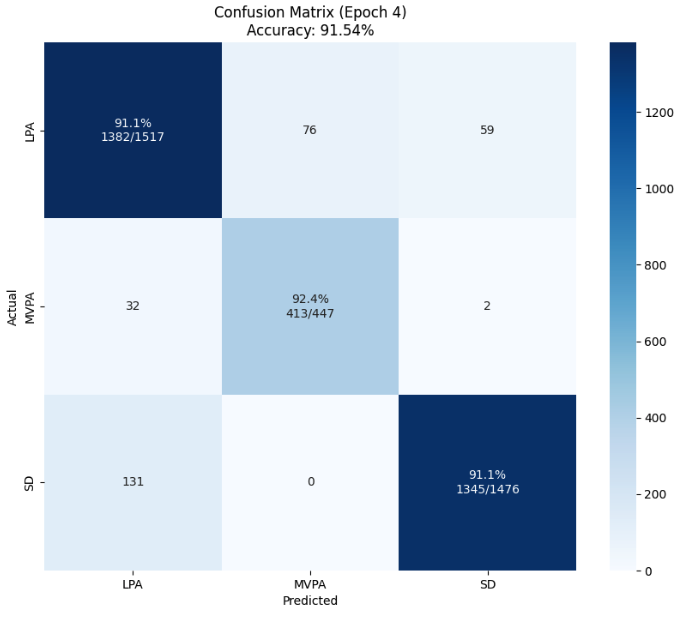


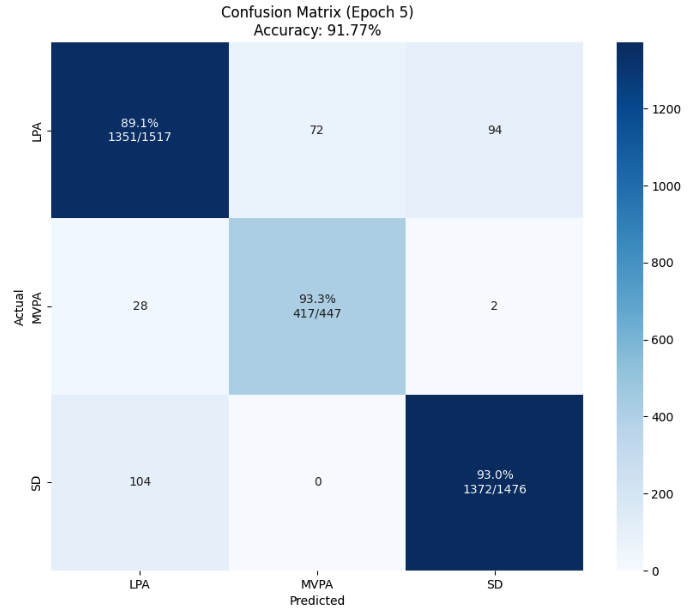


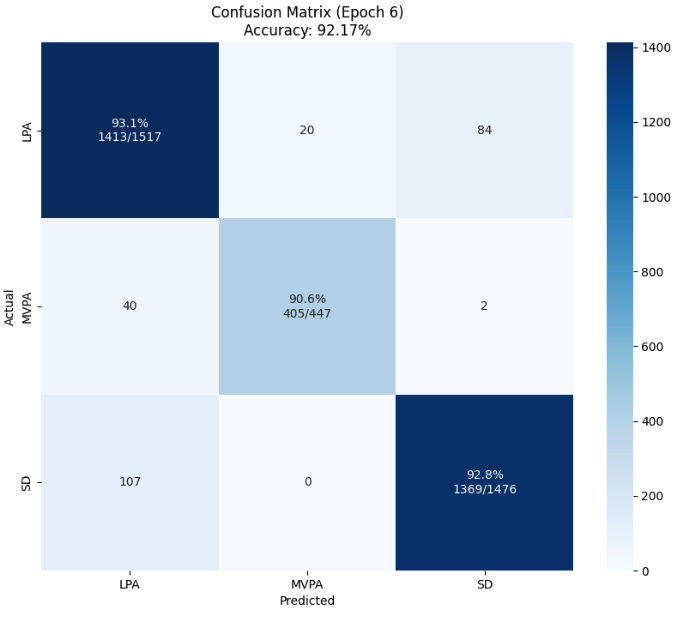


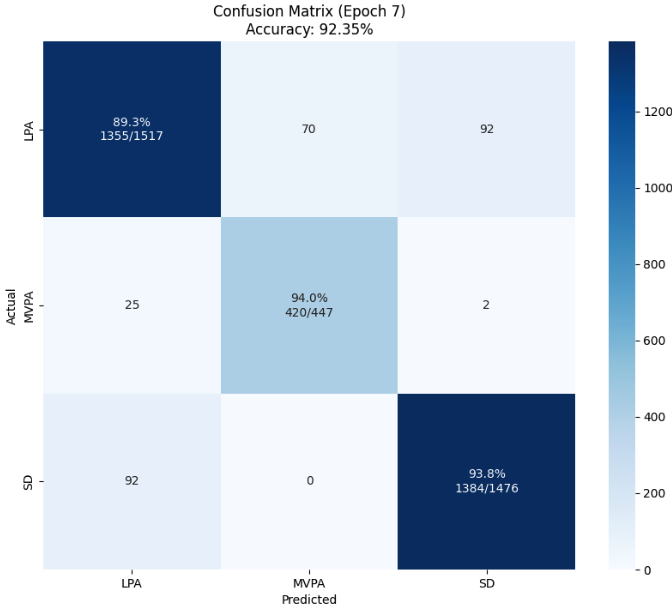


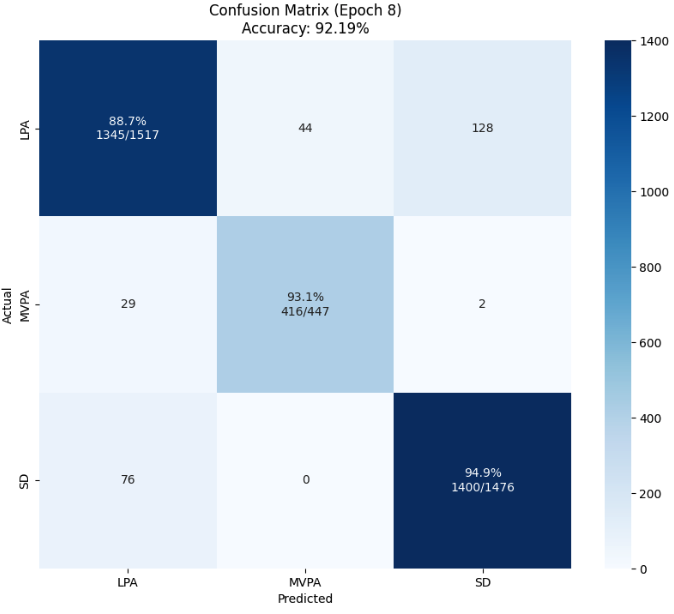


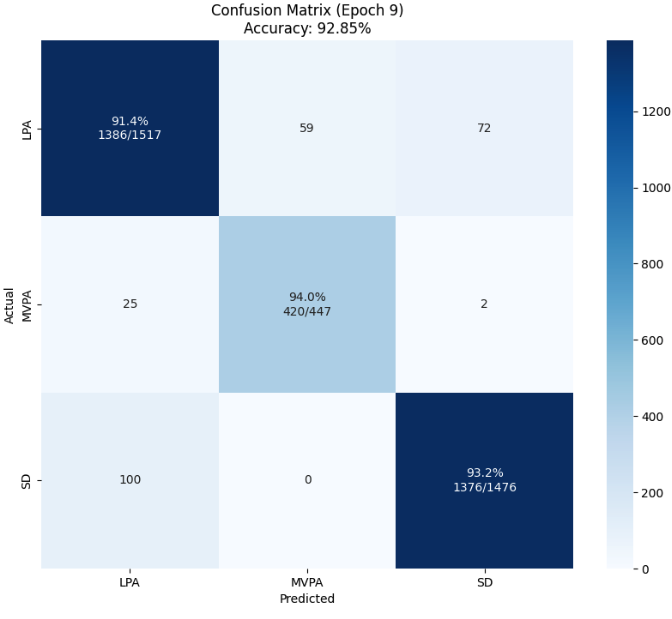


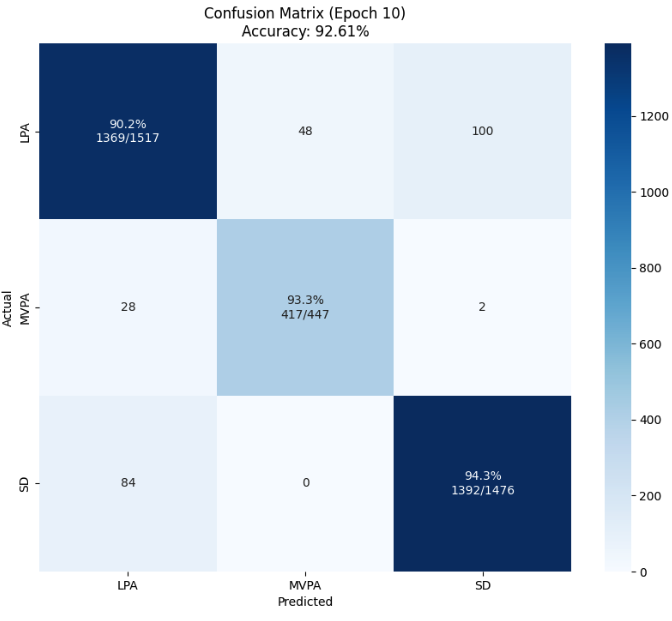


**5 ViT_BiLSTM (METs-based for encoding)**


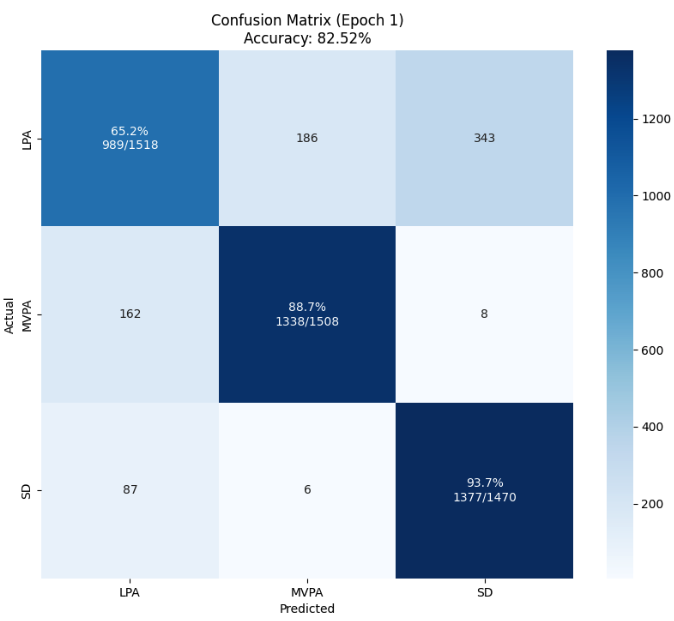


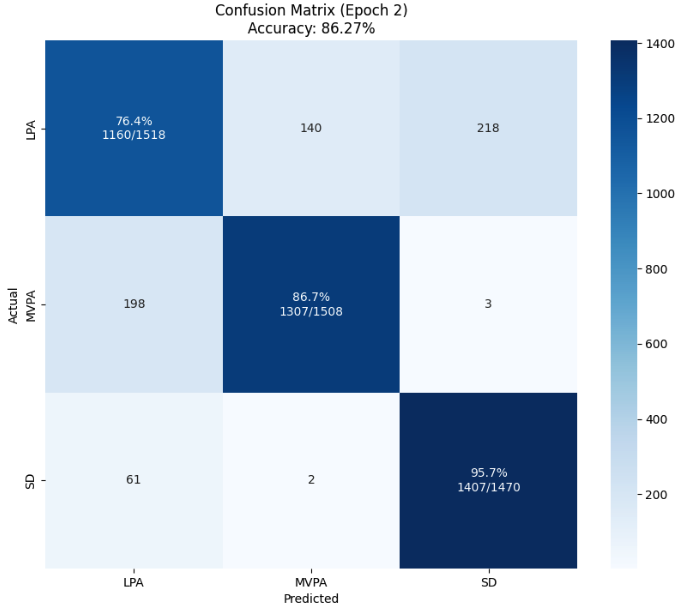


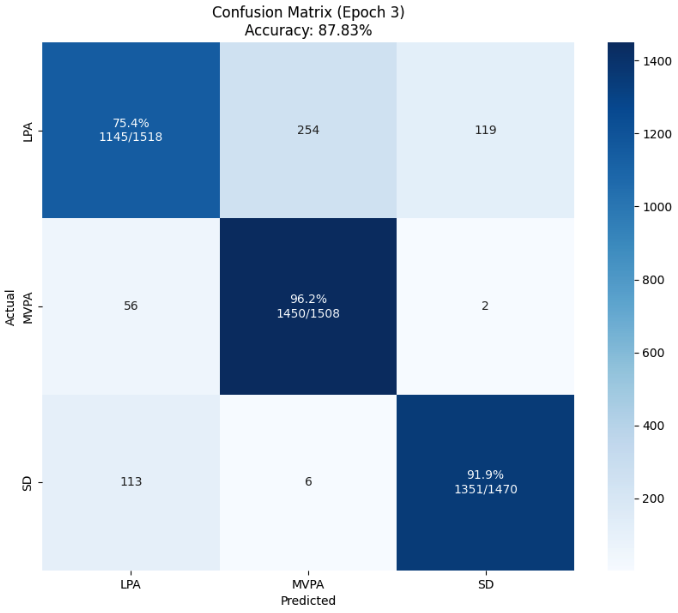


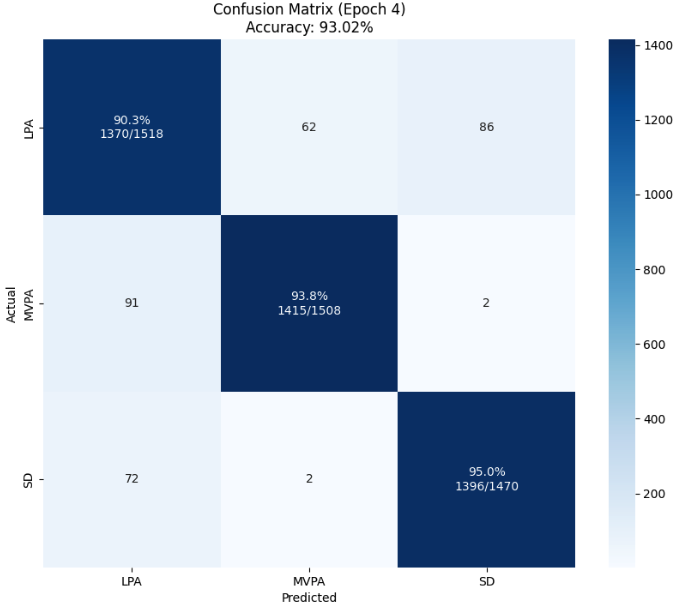


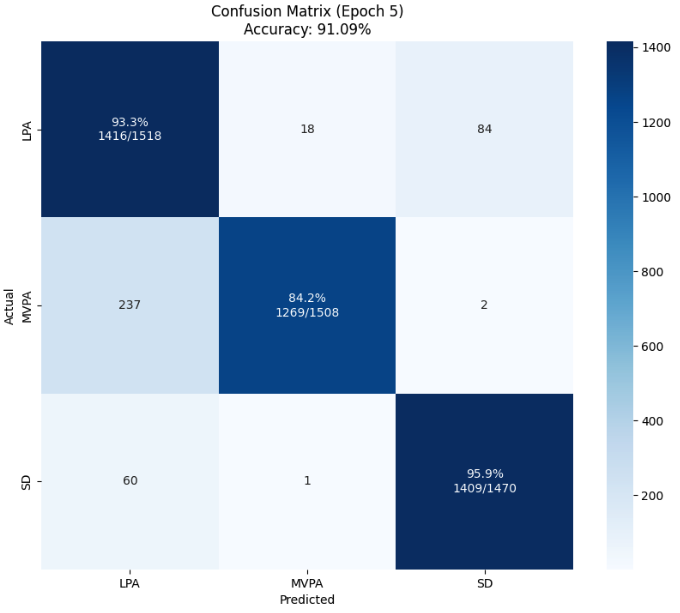


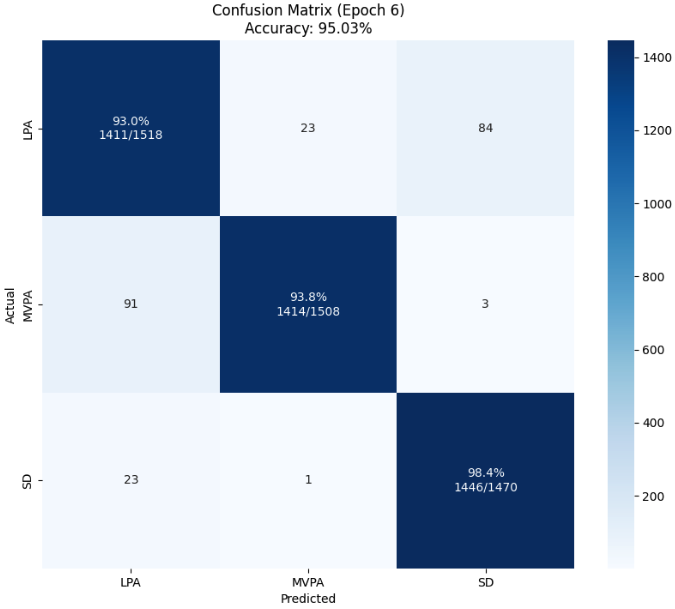


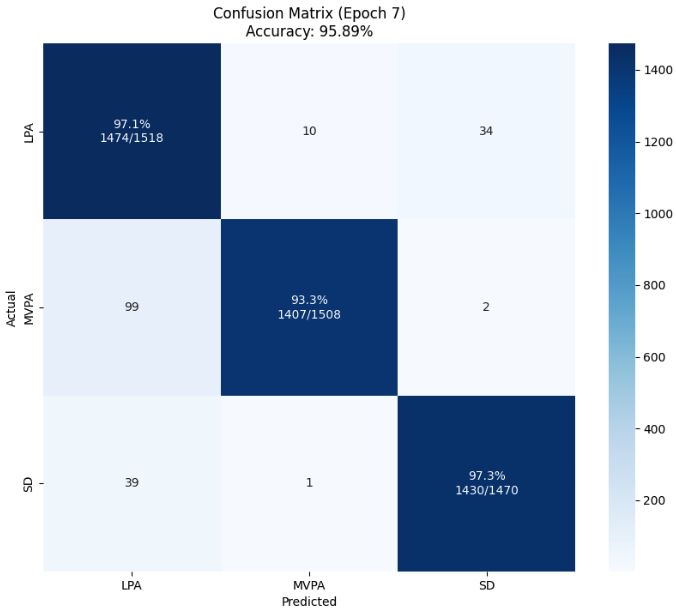


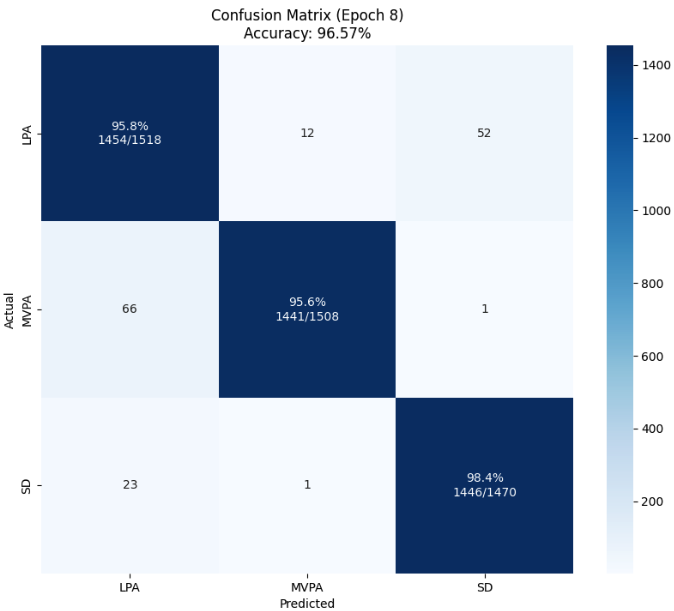


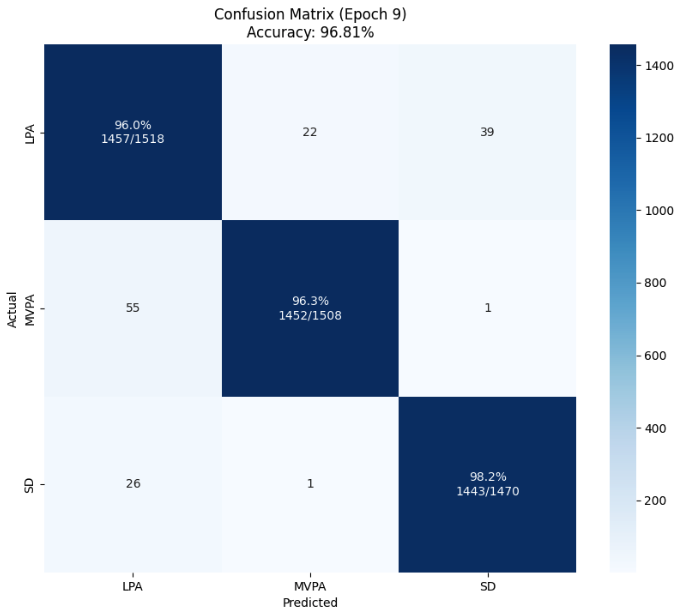


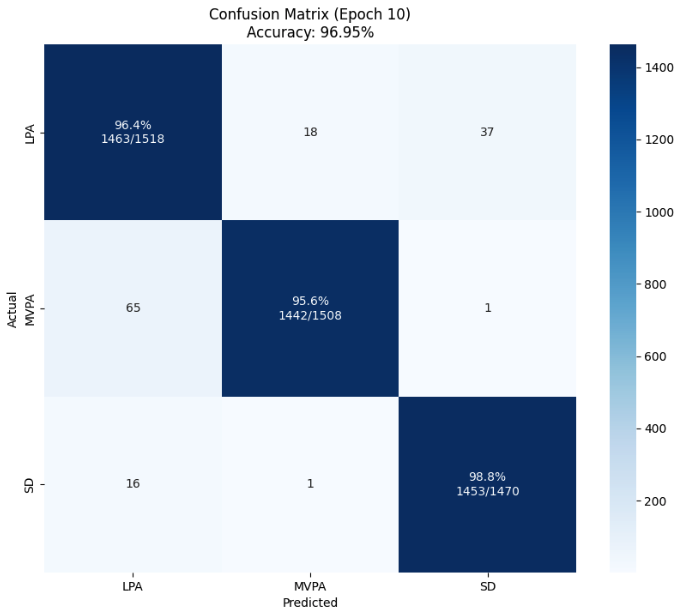

Supplement: Supplementary file 2 — Supplementary Material 2 [file 42490_2025_88_MOESM2_ESM.docx]
