# Supplementary material for: A novel ViT-BILSTM model for physical activity intensity classification in adults using gravity-based acceleration
Source: BMC Biomed Eng. 2025 Feb 1;7:2. doi: 10.1186/s42490-025-00088-2 (PMC11786420; doi:10.1186/s42490-025-00088-2)
Supplement: Supplementary file 3 — Supplementary Material 3 [file 42490_2025_88_MOESM3_ESM.docx]

**Supplementary Material 3**

The results of ANOVA, F-value measures the ratio of variance between the groups to the variance within the groups, indicating whether differences between means are significant. Higher F-values suggest greater between-group differences relative to within-group variation. p-value is that statistical significance was set at p < 0.05. This threshold was chosen following standard practice in machine learning and physical activity research. R-squared (R²) represents the proportion of variance in the dependent variable (accuracy) that is predictable from the independent variables (activity intensity and temporal window). Values range from 0 to 1, with higher values indicating better model fit. Adjusted R-squared: A modified version of R-squared that adjusts for the number of predictions in the model, providing a more conservative estimate of model fit. Root Mean Square Error (RMSE): Measures the standard deviation of prediction errors (residuals), providing an absolute measure of model fit in the same units as the dependent variable. Lower RMSE values indicate better model performance.

Table 1 ANOVA for Model Accuracy across Different Physical Activity Intensities

| Source | Partial SS | df | MS | F | Prob>F | R-squared | Root MSE | Adj R-squared |
| --- | --- | --- | --- | --- | --- | --- | --- | --- |
| Model | 0.004 | 2 | 0.002 | 2.18 | 0.13 |  |  |  |
| Activity | 0.004 | 2 | 0.002 | 2.18 | 0.13 |  |  |  |
| Residual | 0.02 | 27 | 0.001 |  |  | 0.13 | 0.03 | 0.07 |
| Total | 0.03 | 29 | 0.001 |  |  |  |  |  |

Table 2 ANOVA for Model Accuracy across Different Temporal Window

| Source | Partial SS | df | MS | F | Prob>F | R-squared | Root MSE | Adj R-squared |
| --- | --- | --- | --- | --- | --- | --- | --- | --- |
| Model | 0.000 | 4 | 0.000 | 0.52 | 0.72 |  |  |  |
| Activity | 0.000 | 4 | 0.000 | 0.52 | 0.72 |  |  |  |
| Residual | 0.013 | 45 | 0.000 |  |  | 0.04 | 0.01 | -0.04 |
| Total | 0.014 | 49 | 0.000 |  |  |  |  |  |

ANOVA results in Table 1 indicate that the ViT-BiLSTM model's accuracy does not significantly vary across different PAIs (F = 2.18, p = 0.13). This demonstrates the model's stability and reliability across various physical activity classifications. The Table 2 results, the model demonstrated good robustness in predicting across different epoch sizes, with F = 0.52 and p = 0.72. This indicates that the model's performance remains consistent regardless of the variations in TMs.
