# Supplementary material for: A novel ViT-BILSTM model for physical activity intensity classification in adults using gravity-based acceleration
Source: BMC Biomed Eng. 2025 Feb 1;7:2. doi: 10.1186/s42490-025-00088-2 (PMC11786420; doi:10.1186/s42490-025-00088-2)

**Accuracy and Loss Curves for Different Temporal Windows and Models**

This figure shows the accuracy and loss curves for the ViT-BiLSTM model across different temporal windows (1s, 5s, 10s, 15s, 30s), as well as for different models with a 30-second temporal window.

1 Accuracy and Loss Curves for different TWs based on ViT_BiLSTM model

These curves demonstrate the ViT-BiLSTM model's performance in terms of accuracy and loss across different temporal windows

1s


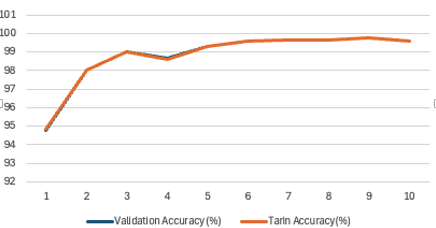

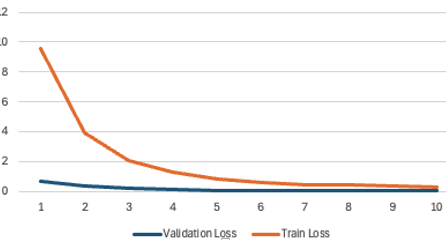


5s


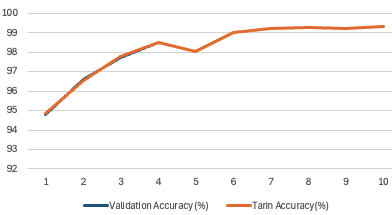

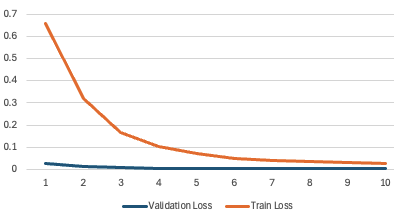


10s


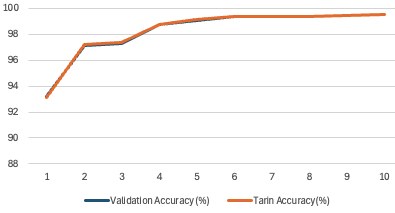

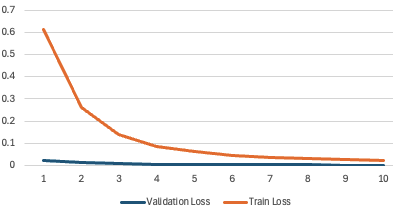


15s


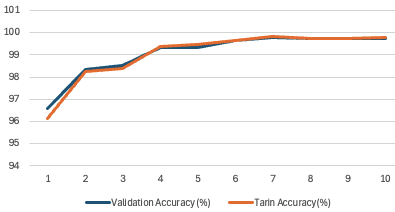

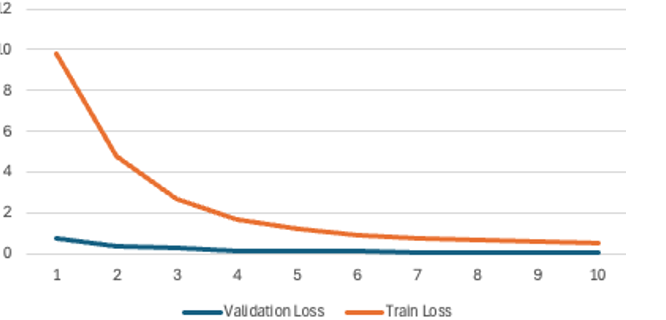


30s


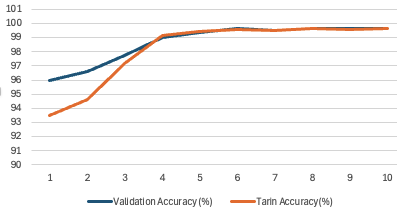

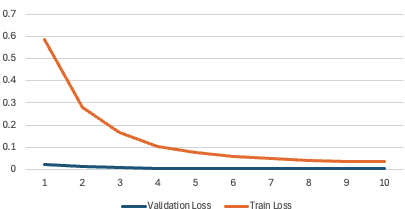


2 Accuracy and Loss Curves for different models with 30 TWs

These curves compare the accuracy and loss for different models, each using a 30-second temporal window for encoding

ViT model (gravity-based encoding)


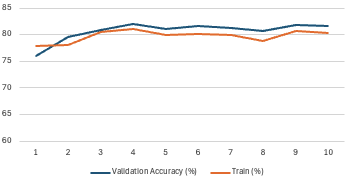

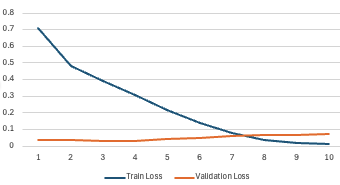


BiLSTM model (Gravity-based encoding)


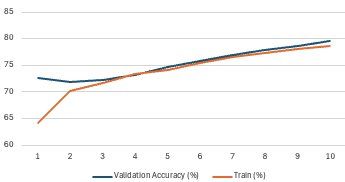

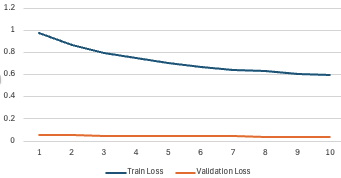


Cnn model (Gravity-based encoding)


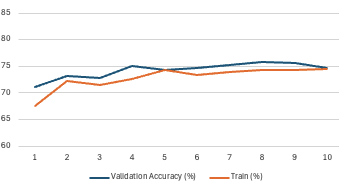

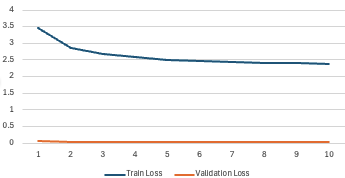


Cnn_BiLSTM model (Gravity-based encoding)


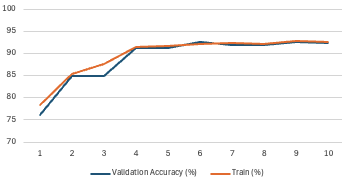

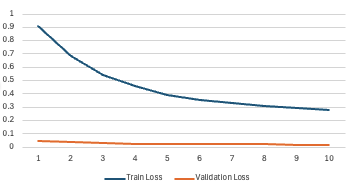


ViT_BiLSTM (METs-based encoding)


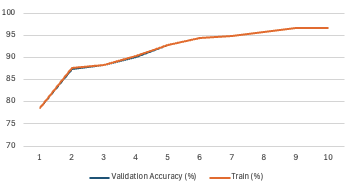

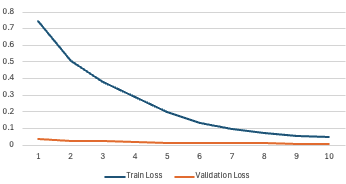


Vit_BiLSTM (gravity-based encoding)


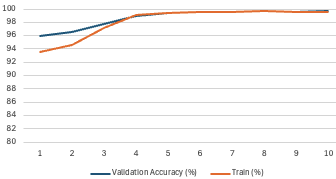

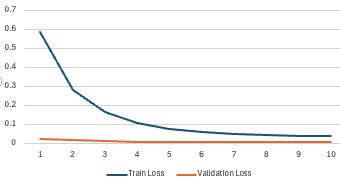

Supplement: Supplementary file 4 — Supplementary Material 4 [file 42490_2025_88_MOESM4_ESM.docx]
