# Supplementary material for: A novel ViT-BILSTM model for physical activity intensity classification in adults using gravity-based acceleration
Source: BMC Biomed Eng. 2025 Feb 1;7:2. doi: 10.1186/s42490-025-00088-2 (PMC11786420; doi:10.1186/s42490-025-00088-2)
Supplement: Supplementary file 5 — Supplementary Material 5 [file 42490_2025_88_MOESM5_ESM.docx]

**Supplementary File Legends**

**Supplementary 1:**

1. The definitions of the x, y, and z axes of Axivity AX3 accelerometer
2. x, y, and z axes in different physical activity intensity.

**Supplementary Material 2:**

Confusion Matrices for Different Models

This figure presents the confusion matrices comparing the performance of different models in classifying physical activity intensities over 10 epochs with a 30-second temporal window.

1. **ViT Model (Gravity-based for Encoding)**

The ViT model uses gravity-based acceleration data to generate images for encoding. The confusion matrix shows the model's classification performance across 10 epochs.

1. **BiLSTM Model (Gravity-based for Encoding)**

The BiLSTM model processes gravity-based acceleration data. The confusion matrix presents the classification results over 10 epochs.

1. **CNN Model (Gravity-based for Encoding)**

The CNN model uses gravity-based acceleration data for image encoding. The confusion matrix displays the classification performance across 10 epochs.

1. **CNN-BiLSTM Model (Gravity-based for Encoding)**

This hybrid model combines CNN for spatial feature extraction and BiLSTM for temporal dependency capturing using gravity-based acceleration data. The confusion matrix shows the results over 10 epochs.

1. **ViT-BiLSTM Model (METs-based for Encoding)**

The ViT-BiLSTM model uses METs-based acceleration data for image encoding. The confusion matrix illustrates the model's performance across 10 epochs.

**Supplementary Material 3:**

**ANOVA results**

1. Table 1 ANOVA for Model Accuracy across Different Physical Activity Intensities
2. Table 2 ANOVA for Model Accuracy across Different Temporal Window

**Supplementary Material 4:**

Accuracy and Loss Curves for Different Temporal Windows and Models

This figure shows the accuracy and loss curves for the ViT-BiLSTM model across different temporal windows (1s, 5s, 10s, 15s, 30s), as well as for different models with a 30-second temporal window.

1. **Accuracy and Loss Curves for Different Temporal Windows Based on ViT-BiLSTM Model**

These curves demonstrate the ViT-BiLSTM model's performance in terms of accuracy and loss across different temporal windows:

- - - 1 second
    - 5 seconds
    - 10 seconds
    - 15 seconds
    - 30 seconds

1. **Accuracy and Loss Curves for Different Models with 30-Second Temporal Window**

These curves compare the accuracy and loss for different models, each using a 30-second temporal window for encoding:

- - - ViT model (Gravity-based encoding)
    - BiLSTM model (Gravity-based encoding)
    - CNN model (Gravity-based encoding)
    - CNN-BiLSTM model (Gravity-based encoding)
    - ViT-BiLSTM model (METs-based encoding)
    - ViT-BiLSTM model (Gravity-based encoding)
